# The Monongahela tradition in “real time”: Bayesian analysis of radiocarbon dates

**DOI:** 10.1371/journal.pone.0276014

**Published:** 2022-10-26

**Authors:** John P. Hart, Bernard K. Means

**Affiliations:** 1 Research and Collections Division, New York State Museum, Albany, New York, United States of America; 2 School of World Studies, Virginia Commonwealth University, Richmond, Virginia, United States of America; Austrian Academy of Sciences, AUSTRIA

## Abstract

Despite advances in techniques, methods, and theory, northeastern North American archaeologists continue to use early to mid-twentieth century culture historical taxa as units of analysis and narrative. There is a distinct need to move away from this archaeological practice to enable fuller understandings of past human lives. One tool that enables such a move is Bayesian analysis of radiocarbon dates, which provides a means of constructing continuous chronologies. A large dataset of radiocarbon dates for late prehistoric (ca AD 900/1000–1650) sites in the lower upper Ohio River basin in southwestern Pennsylvania and adjacent portions of Maryland, Ohio, and West Virginia is used here as an example. The results allow a preliminary assessment of how the settlement plans of contemporaneous villages varied considerably, reflecting decisions of the village occupants how to structure built environments to meet their needs.

## Introduction

Contemporary eastern North American archaeologists have a stark choice. They can either practice archaeology or they can use the archaeological record to build knowledge of how people lived in the past. By practicing archaeology, we mean imposing early to mid-twentieth-century culture-historical taxa on the past as units of analysis and narrative. The problems with this approach have been delineated in both regional analyses [1–4] and more recent general critiques [5, 6]. Taxonomic methods were first developed at a time when there was no appreciation for the time depth of Indigenous occupations in North America (e.g., [7], p.10, [8], p. xxvii) and when there was no means independent of archaeological artifact analysis, and in some instances stratigraphy, to determine relative chronologies [9]. The primary purpose of culture-historical taxa was to allow archaeologists to control for chronological and spatial variations in artifact distributions [10, 11]; they were merely categorization units developed at a time when archaeological tool kits were very limited [12]. Despite advances in methods, techniques, and theory since then that allow the development of chronologies independent of artifactual analysis [13–16] and improvements in methods for building understandings of regional and interregional interactions [17–19] and movements [20, 21], many archaeologists in eastern North America continue to use culture-historical taxa as units of analysis and narrative. Culture historical taxa are enmeshed in university level textbooks (e.g., [22, 23]) and contemporary peer-reviewed literature (e.g., [24–30]). Their use, however, is an archaeological practice, having little to do with how people lived in the past. Simply put, culture-historical methods, designed to limit variation within taxa, prevent archaeologists from understanding the full range of variation in past human lifeways; variation is emphasized only at chronological and spatial taxonomic boundaries [31]. Given their entrenchment within the discipline, simply assigning a site or sites to a taxon or taxa prior to any subsequent analyses establishes expectations for how the site(s) fit within long-established regional archaeological narratives. As a result, archaeologists’ abilities to contribute meaningfully to understandings of spatial and chronological variation in past Indigenous lifeways outside of the framework of culture history has been limited.

Assumptions about how artifacts change through time have guided archaeological approaches to chronology building for generations [9, 32]. Ideally, site-level relative chronologies were built combining artifact analyses and stratigraphic contexts (percentage stratigraphy) and extended to multiple sites within a region (interdigitation) to construct relative chronologies [9]. In situations where sites lacked stratigraphy, frequency seriation of types from multiple sites in a region relied on the so-called popularity principle [9, 32]. This in turn was used to help order culture-historical taxa in relative chronological order. The advent of radiocarbon dating in the 1950s based on physical laws and theory provided an independent means of estimating the actual dates of site occupations once samples are chosen for assay [16]. However, radiocarbon dating was generally simply added to culture-historical methods as a means to anchor taxa in time (e.g., [8, 33]). It continues to be used in many areas to determine dates for taxonomic boundaries and site placements within those boundaries (e.g., [34]) including with contemporary Bayesian chronological modeling (e.g., [24, 35]).

One such region is the lower upper Ohio River basin in what is now southwestern Pennsylvania and immediately adjacent portions of present-day Maryland, Ohio, and West Virginia. Archaeological sites dating to approximately the mid-eleventh to mid-seventeenth centuries AD in this region are generally assigned to the Monongahela tradition or culture, named after one of the rivers forming the Ohio River at present-day Pittsburgh. Originally described in the 1930s based on three sites in the Allegheny Mountains east of Pittsburgh [36], per extensional definition practice [31], the taxon was revised to encompass the larger lower upper Ohio River basin as additional sites were identified and excavated [37]. Mayer-Oakes [37] established a periodization scheme for the Monongahela taxon consisting of Early, Middle, and Late periods that with modifications is still used today as a method for controlling time (e.g., [38]). Archaeologists working in the lower upper Ohio River basin have regularly obtained radiocarbon dates from late prehistoric sites to assist in refinements of Mayer-Oakes’ periodization scheme.

There has been a general narrative that certain Monongahela tradition subsistence and settlement traits changed through time. Hart and colleagues [39] challenged this narrative by demonstrating that most of the traits did not exhibit the postulated changes. Rather, there was continuous regional variation in the traits except for narrow, enclosed structures attached to circular houses, which increased in frequency through time. However, like previous attempts to track changes in Monongahela tradition subsistence-settlement traits through time independent of culture historical time periods [40, 41], this analysis was constrained by limited numbers of radiocarbon dates both at the site and regional levels (see [42]). Consequently, there was a component of subjectivity in the placement of sites in chronological sequence.

It was not until Means’ [43–46] large-scale dating project for sites excavated in the 1930s by Works Project Administration crews in the Allegheny Mountains that radiocarbon dating began to realize its potential for chronology building in the region independent of culture-historical taxa. Unfortunately, none of the three sites used to originally define the Monongahela tradition by Butler [36] had available collections suitable for radiocarbon dating, and the excavation data on the sites themselves were problematic [47]. However, while traditionally assigned to the Early Monongahela period because of the use of crushed limestone- as opposed to shell-tempered pottery [48], Means demonstrated that some sites in the Allegheny Mountains region were occupied well into Mayer-Oakes’ Middle Monongahela period as currently conceived. Since Means’ project, chronology building at the site and regional levels in general has been enhanced by developments in Bayesian analysis of radiocarbon dates [13, 49] and various means of combining and summarizing large sets of radiocarbon dates (e.g., [50–52]). However, these methods and techniques have not been adopted by archaeologists working in the lower upper Ohio River basin. The most active use of Bayesian analysis in northeastern North America has been in fourteenth through mid-seventeenth-century AD contexts in the northern Iroquoian region of New York, Ontario, and Québec (e.g., [15, 53–58]). These analyses have shown in many cases that chronological placements of sites based on traditional archaeological practice can be substantially in error.

In an earlier assessment of all known radiocarbon assays from sites attributed to the Monongahela tradition, Means [42] noted that of approximately 400 radiocarbon assays, only a few sites had more than one radiocarbon assay, and, even if sites had more than one assay, they were often of a questionable nature. Among the issues were problems with sample selection and a failure to link material selected for dating to specific contexts. This was notable at the Gnagey No. 3 site (36So55), which was originally dated by George [48] on the basis of several radiocarbon dates. AMS radiocarbon dates obtained later on specimens from the site’s collection by Hart and Scarry [59] and Means [43, 44] produced a chronology substantially different from that outlined by George [48]. Means [42] also found that the geographic distribution of dated Monongahela tradition sites was uneven and concentrated in areas with major CRM investigations, such as the Meyersdale Bypass Project in the Allegheny Mountains section; university-led research, especially by Indiana University of Pennsylvania in the Allegheny River basin north of Pittsburgh; and targeted dating of legacy collections [43, 44, 46, 59, 60]. Maps showing the spatial boundaries of different Monongahela tradition phases by Johnson and Means [38] were largely created absent a connection to dated components. In their recent review of the Monongahela tradition, Johnson and Means ([38] p. 370) defaulted to Mayer-Oakes’ three-time-period scheme and its associated phases to review “Changes and Evolution in Adaptive Strategies” recognizing the need for “a more refined chronological framework” through more expansive and targeted programs of radiocarbon dating.

To begin to address this need and further demonstrate the potential of Bayesian modeling of radiocarbon dates for regional chronology building in the Northeast for the span of time in question, Means’ earlier work is expanded here through application of Bayesian modeling to large sets of radiocarbon dates obtained over the past several decades by site investigators working throughout the Monongahela tradition region. This method makes it possible to model Indigenous history continuously rather than in subjective blocks of time. While we acknowledge the limitations of the existing database of radiocarbon dates, the results allow us to begin to understand variation in how Indigenous communities adapted their build environments to changing local circumstances. The results also emphasize the need for many additional radiocarbon dates in order to fully model the region’s chronology.

### The Monongahela tradition

**Fig 1:**
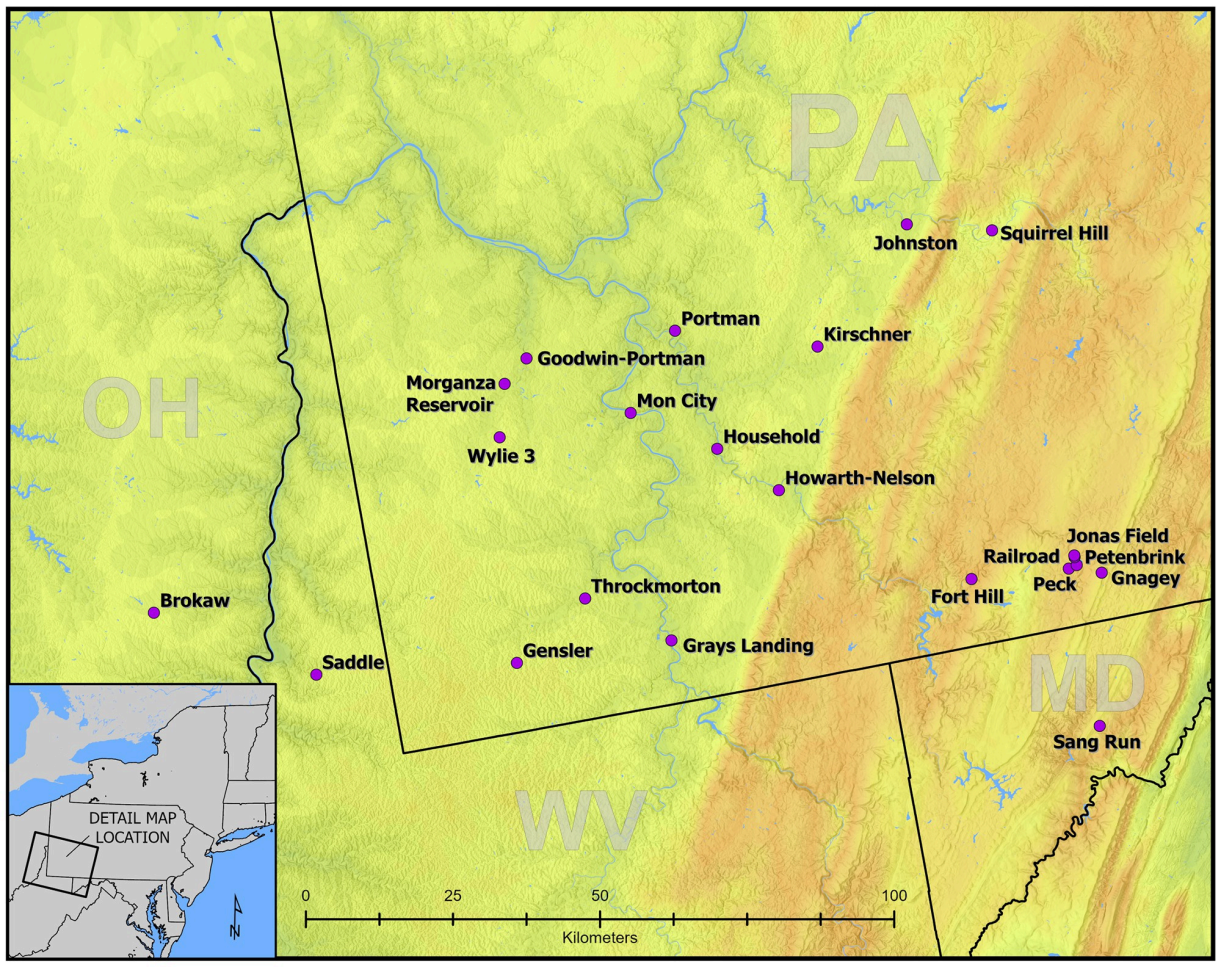
Locations of sites with radiocarbon dates used in the Bayesian modeling. This map was produced in ArcGIS v 10.6 at the New York State Museum in Albany by compiling GIS shapefiles obtained from publicly available sources including Statistics Canada, the United States Census, and the United States Geological Survey.

The Monongahela tradition is an archaeological taxon encompassing sites in the lower upper Ohio River basin of southwestern Pennsylvania and adjacent portions of Maryland, Ohio, and West Virginia that date to ca. AD 1050–1635 (Fig 1) [38]. As previously mentioned, three time periods are generally defined for the tradition, Early (AD 1050/1100–1250), Middle (AD 1250–1590), and Late (AD 1590–1635). Time period boundaries are primarily based on changes in pottery assemblages between the Early and Middle periods and the first occurrence of European trade goods between the Middle and Late periods [38]. Sites are sometimes denoted as early or late within the lengthy Middle Monongahela period. Radiocarbon assays obtained from legacy collections and CRM investigations for Monongahela tradition sites in Allegheny Mountains section of the Appalachian Plateau Province demonstrated that these villages do not fit into the traditional chronological framework based on pottery. Johnson and Means [38] assigned these sites to a Somerset sub-tradition—as most of the sites are in Somerset County, Pennsylvania—which was arbitrarily divided into Somerset I and Somerset II sub-traditions at the Early and Middle Monongahela boundary. The procrustean manipulations used to create the Somerset sub-tradition is at least tacit recognition that the traditional pottery-based Monongahela temporal framework is increasingly untenable in the face of an expanding radiocarbon database.

The Monongahela tradition is typified in the archaeological literature by circular-to-oval villages consisting of small, round houses placed in rings around central plazas [38, 46]. These plazas may contain large central posts, fire pits, or evidence of intense burning. Most, but not all, of these villages were surrounded by apparently insubstantial stockades, perhaps used more to delineate the boundaries of the villages than to serve defensive purposes. Rather than deep storage pits, which occur in many areas of contemporaneous temperate northeastern North America regions [61], what are generally interpreted as storage facilities consist of shallow, often elongated pits surrounded by postmolds, which presumably represent aboveground structures. House patterns often have one or more of these facilities attached with openings from within the house for access, although they also occur adjacent but not attached to house patterns. Large, circular structures late in the sequence may have many such appendages and have been designated as “petal structures.” Also occurring on such sites may be so-called charnel houses—circular structure patterns within which multiple human pit burials occur. Smaller domestic sites, often referred to as hamlets, consisting of a few houses also occur. Stable carbon isotope values on human bone collagen and tooth caries indicate diets could include large proportions of maize. Pottery is typically tempered with crushed limestone or shell with plain or cordmarked surfaces and minimal decoration. Collars are present on ceramic jars in some late assemblages.

Narratives of the Monongahela tradition often posit chronological changes in these subsistence and settlement traits. Johnson et al. [62] and Johnson [63] argued that there were increases in village size starting with the Middle Monongahela period reflecting territorial contraction and deterioration of climatic conditions. They also concluded there was a decrease in village plaza size occurred in the Late Monongahela period compared to the Early and Middle periods. Means [46], however, found that villages in the Allegheny Mountains did not indicate a correlation between a village’s size and its age during the Early and Middle Monongahela periods. Hasenstab and Johnson [64], Johnson [65], and Johnson et al. [62] speculated that there was abandonment of locations with growing seasons ≤140 days at the beginning of the Middle Monongahela period as a result of changes in climate detrimental to maize production. George [48, 66] concluded that there was an early movement to upland settings as defense against internecine warfare, while Johnson [65] saw such a movement in the Late Monongahela period resulting from hostile incursions from the west. Several studies indicated increases in the percentages of houses with attached storage structures beginning with the Middle Monongahela period [40, 42, 62, 65, 67]. Nass and Hart [42] found continuous variation in household size based on house floor area throughout the sequence. Finally, maize consumption was posited to have increased through time (e.g., [40, 41, 67, 68]).

A previous analysis tested these posited trends as hypotheses [39]. Based on 155 radiocarbon dates available at the time, 61 sites and site components were placed in approximate chronological order using means of the available radiocarbon dates independent of the culture-historical time periods. Statistical analyses indicated that all but two of the hypotheses, household size variation and increases in percentages of houses with attached storage facilities, could be rejected. As a result, it was concluded that Mayer-Oakes’ [37] time periods were unhelpful in building understandings of the people who created the record identified by archaeologists as the Monongahela tradition. Means’ [46] exhaustive analysis of settlement patterns of Monongahela tradition villages in the Allegheny Mountains in conjunction with a large-scale radiocarbon dating effort further undermined traditional narratives on the Monongahela tradition. According to Means ([46], p. 145), the “village sites clearly exhibit variation in their spatial layouts and their constituent social groups. Accounting for this variation cannot be achieved by examining individual components in isolation.”

Since these analyses there has been a substantial increase in the number of radiocarbon dates from the region. Our goal here is to demonstrate their potential to create a chronology entirely independent of culture-historical taxa as a foundation for enhancing existing and building new knowledge about how people in this region lived their lives in the centuries prior to disruptions and dislocations resulting from European entrance and persistent presence in eastern North America. This approach will ultimately allow us to begin to expand Mean’s [46] earlier work to the larger region as additional sites are radiocarbon dated for the first time and additional dates are obtained from inadequately dated sites.

## Methods and materials

No permits were required for the described study, which complied with all relevant regulations. A compilation of radiocarbon measurements that includes previously published dates, dates uploaded to the Canadian Archaeological Radiocarbon Database (CARD) [69], and previously unpublished dates from two sites were used in the following analyses. Only samples taken from secure feature contexts were used in the analyses. Radiocarbon measurements on mollusk shell and mollusk shell temper were not included in the models because of the likelihood for freshwater reservoir offsets. Radiocarbon measurements obtained from the Gakushuin Laboratory (Gak) in the early 1980s were not included in the models because of reliability issues [70, 71]. All radiocarbon measurements and associated data are provided in S1 Table. Bayesian modeling was performed with OxCal v. 4.4.4 [13] using the IntCal20 terrestrial Northern Hemisphere calibration curve [72]. Three Outlier Models were used in the modeling ([49, 73]: “Charcoal” for dates on wood charcoal, “SSimple” for dates on samples from same feature and splits of the same sample that are combined with the R_Combine code, and “General” for the resulting combined date and for dates on all other materials other than charcoal. OxCal run files for all models are provided in S1 Appendix. All OxCal terms are capitalized in the text to differentiate them from more common uses of the terms in archaeology.

Each site or site component was modeled as a Phase. The Date command was used to generate a hypothetical event that summarizes knowledge of the Phase as inferred from the Start and End Boundaries ([50], p. 1812; see [7, 15, 54, 56, 57, 74]). In cases where specific sites had multiple village patterns or available dates suggest multiple occupations, a sequential Phase model was run for each site to determine if hiatuses are probable between village occupations. If the models resulted in Interval ranges between components containing 0 and/or the Date estimates overlapped, this suggested the probability of continuous rather than discrete occupations of the sites. If two or more occupations were not chronologically discrete based on this criterion, then all dates for the site were combined in a single Phase in multi-Phase models. If the presumed separate occupations were chronologically discrete, as indicated by the lower value of Interval estimates being >0 and/or Date estimates not overlapping, each occupation was treated as a separate Phase in the multi-Phase models. Overlapping Phase models were run for two geographical distinct subregions of the upper lower Ohio River basin: sites in the Allegheny Mountains and sites on the Unglaciated Allegheny Plateau west of Chestnut Ridge (Fig 1).

Because the spans of time in the subregional models are extensive, they encounter several reversals/plateaus in the calibration curve resulting in multi-modal highest posterior densities (hpd). To address this issue, following [7] the log-normal distribution for site Interval constraints with the command Interval(“”,LnN(ln(50,ln(2))). Alternate models were run with normal distribution Interval constraints with the command Interval(“”,N(50,10)). This was done under the assumption that like village sites in other regions of northeastern North America [75, 76], occupation spans were relatively short, lasting for a few to several decades before a village was abandoned with the community moving to a newly constructed village some distance away. These constraints are overridden if the radiocarbon dates indicate longer occupations ([77], p.6). For consistency, the constraints were applied to all Phases in the subregional models even when a site’s dates did not fall on a reversal/plateau.

Models were accepted if the Agreement indices (model and overall) were ≥60% and all model elements converged at ≥95%. Interval constraints <50 years failed to meet these criteria. Including the Johnston site, for which a very large number of dates are available, with or without Interval constraints in the overlapping Unglaciated Plateau models prevented some components of models from converging at ≥95%. Modeling the site separately and including Interval constraints resulted in specific dates and Interval and Date estimates from converging at ≥95%. As a result, Johnston was modeled separately as a uniform Phase without Interval constraints, which resulted in all components of the models converging at ≥95% and agreement indices ≥60%.

Date estimate and Start boundary posterior probability raw data were saved for each Phase in each model. These data were then used in separate models with the Order command, which produced a matrix of order probabilities for all Phases from both subregional models. The unconstrained Johnston model was included in each of the models.

## Results

### Unglaciated Allegheny Plateau

The Campbell Farm, Consol, Kirschner, and Saddle sites have indications of two or more occupations based on site plans or what appear to be discrete groupings of radiocarbon ages. Consol has been completely excavated and two slightly overlapping village patterns were documented [78]. Radiocarbon dates suggest the larger village is later in time than the smaller village. Feature 245, which fell within the larger of the two villages, produced the earliest dates from the site suggesting an older occupation is present within the area of the large village pattern. A charnel house within the pattern of the larger village was assumed to be a component of that village and the dates obtained from it were included in the late Phase of the model. Campbell Farm [64] and Kirschner [65] have been partially excavated and it appears that the occupations at each site overlap or are superimposed. Saddle has been interpreted as two spatially distinct components, an early hamlet consisting of two structures and later village [79]. Each of the sites was modeled separately with Sequential Phases, and using the normal-distribution Interval constraint (N(50,10)) to determine if there were hiatuses between occupations or if there was a single occupation that accounts for the date range. Results suggest there were hiatuses between the occupations at all the sites except the early and late occupations at Consol (Table 1) with intervals between occupations ranging from a several decades to over a century (Kirschner) based on 63.8% hpd. As a result, each occupation at the sites was included separately in the regional models. Because the two occupations at Consol represent discrete village patterns and the potential total length of the occupations, the early and late occupations at Consol were also included separately in the regional models.

**Table 1:** Date estimates of and intervals between site-specific sequential Phases in southwestern Pennsylvania south of the Ohio River.

Site Component	Date Estimate (64.3% hpd)	Date Estimate (95.4% hpd)	Interval Between (68.3% hpd)	Interval Between (95.4% hpd)	Agreement Indices	
					model	overall
Campbell Farm early	*1245–1281*	*1226–1297*	98–128	78–144	127.8	128.0
Campbell Farm late	*1404–1440*	*1388–1456*				
Consol F-265	*1221–1271*	*1196–1295*	18–86	0–107	108.0	108.2
Consol early	*1323–1389*	*1288–1400*				
			0–49	0–87		
Consol late	*1414–1453*	*1396–1472*				
Kirschner early	*1278–1309*	*1264–1324*	113–147	96–161	144.4	144.3
Kirschner late	*1448–1486*	*1430–1505*				
Saddle early	*1184–1252*	*1149–1290*	42–121	8–163	89.9	92.2
Saddle late	*1317–1379*	*1298–1415*				

No prior information exists on specific sequences of sites in this region other than multiple occupations at specific sites. Researchers were often content with placing sites in cultural-historical periods without considering their placement within the periods, such as in the discussion of the Scarem phase by Johnson and Means ([38], p. 378). As a result, an overlapping multi-Phase model [13] was developed for 25 sites/site components using the log normal and normal distribution interval constraints. All villages and small habitation sites/hamlets in the region with at least two dates that were not identified as outliers were included in the models, except Johnston as noted previously. Results of the models are presented in Table 2. All elements of the models converged at ≥95%. All elements of the uniform Phase model with no Interval constraint for the Johnston site converged at ≥95%. Agreement indices for all models were ≥60%. The results of the log-normal and normal-distribution models are in accord, with the log-normal distribution Interval constraint model results generally being somewhat less constrained than the normal distribution Interval constraint model.

**Table 2:** Start boundaries, Date estimates, and End boundaries for sites/site components west of Chestnut Ridge.

Site/Component	Model	n	Culture-Hist. Time Period	68.3% hpd			95.4% hpd		
				Start Boundary	Date Estimate	End Boundary	Start Boundary	Date Estimate	End Boundary
Grays Landing 1	1	4	Early	*1149–1224*	*1170–1252*	*1197–1274*	1042–1088 (6.4) 1106–1257 (89.1)	*1072–1285*	*1089–1142* (7.0) *1164–1305* (88.5)
	2	4		*1141–1226*	*1165–1250*	*1190–1275*	*1045–1254*	*1066–1281*	*1079–1131* (6.1*) 1145–1310* (89.4)
Consol Feature 245	1	3	Early	*1183–1223*	*1204–1253*	*1233–1270*	*1163–1248*	*1179–1280*	*1220–1299*
	2	3		*1171–1233*	*1201–1256*	*1229–1269*	*1109–1258*	*1151–1286*	*1217–1317*
Saddle Early	1	4	Early	*1162–1225*	*1184–1254*	*1212–1276*	*1124–1264*	*1142–1295*	*1175–1314*
	2	4		*1155–1226*	*1179–1251*	*1206–1276*	*1054–1073* (1.6) *1096–1264* (93.9)	*1075–1079* (0.2) *1123–1299* (95.2)	*1166–1332*
Drew	1	2	Early	*1186–1256*	*1207–1282*	*1235–1305*	*1141–1286*	*1161–1317*	*1191–1335*
	2	2		*1179–1259*	*1204–1281*	*1227–1304*	*1118–1291*	*1149–1325*	*1180–1354*
Campbell Farm Early	1	4	Early	*1221–1248*	*1240–1280*	*1274–1294*	*1208–1258*	*1220–1295*	*1260–1310*
	2	4		*1216–1255*	*1240–1281*	*1271–1298*	*1180–1265*	*1207–1301*	*1243–1325*
Kirschner Early	1	9	Early	*1266–1285*	*1280–1310*	*1307–1326*	*1255–1290*	*1267–1325*	*1300–1337*
	2	9		*1273–1289*	*1283–1305*	*1299–1316*	*1260–1295*	*1270–1321*	*1291–1335*
Wylie 3	1	5	Early	*1251–1295*	*1270–1325*	*1301–1344*	*1225–1334*	*1244–1364*	*1279–1391*
	2	5		*1244–1297*	*1267–1325*	*1291–1348*	*1192–1332*	*1234–1380*	*1275–1413*
Morganza Reservoir	1	3	Early?	*1251–1306*	*1274–1335*	*1302–1355*	*1232–1358*	*1250–1385*	*1285–1409*
	2	3		*1234–1305*	*1266–1340*	*1298–1365*	*1182–1365*	*1226–1400*	*1282–1430*
Ashmore Farm	1	8	Early/Middle	*1295–1335*	*1315–1367*	*1340–1385*	*1286–1378*	*1300–1406*	*1335–1422*
	2	8		1300–1343	*1313–1365*	*1334–1375* (59.5) *1400–1410* (8.8)	*1285–1359* (86.0) *1367–1392* (9.4)	*1303–1407*	*1325–1425*
Consol Early	1	3	Early	*1282–1295* (13.0) *1325–1368* (55.2)	*1327–1397*	*1330–1342* (9.7) *1372–1416* (58.6)	*1270–1378*	*1285–1410*	*1317–1428*
	2	3		*1277–1301* (22.6) *1331–1373* (45.7)	*1301–1323* (13.6) *1340–1395* (54.7)	*1323–1335* (6.7) *1365–1417* (61.6)	*1258–1382*	*1278–1415*	*1310–1438*
Gensler	1	3	Early	*1281–1317* (28.4) *1331–1377* (39.8)	*1312–1399*	*1330–1361* (24.5) *1377–1425* (43.7)	*1264–1394*	*1281–1426*	*1312–1444*
	2	3		*1279–1323* (38.1) *1343–1380* (30.1)	*1300–1340* (29.6*) 1352–1399* (38.7)	*1319–1350* (22.0) *1370–1421* (46.3)	*1254–1399*	*1276–1426*	*1301–1450*
Portman	1	3	late Middle	*1322–1375*	*1339–1400*	*1370–1423*	*1280–1385*	*1300–1421*	*1330–1438*
	2	3		*1312–1378*	*1334–1402*	*1362–1429*	*1262–1389*	*1285–1436*	*1318–1467*
Goodwin-Portman	1	3	Middle	*1301–1386*	*1324–1412*	*1349–1435*	*1271–1413*	*1290–1444*	*1322–1463*
	2	2		*1303–1399*	*1330–1426*	*1355–1453*	*1254–1439*	*1286–1472*	*1317–1501*
Saddle Late	1	6	Middle	*1313–1368*	*1335–1396*	*1364–1419*	*1294–1393*	*1311–1426*	*1343–1443*
	2	6		*1307–1365*	*1330–1392*	*1354–1417*	*1285–1400*	*1305–1428*	*1331–1452*
Morganza-Lang	1	2	Middle	*1283–1315 (24*.*9) 1325–1378 (43*.*4)*	*1311–1401*	*1329–1360 (23*.*2) 1374–1426 (45*.*1)*	*1258–1402*	*1275–1435*	*1307–1452*
	2	2		*1280–1325* (36.0) *1340–1381* (32.3)	*1302–1342* (28.4) *1350–1401* (39.8)	*1319–1354* (22.3) *1367–1424* (46.0)	*1243–1407*	*1270–1438*	*1298–1462*
Howarth-Nelson	1	5	Middle	*1382–1415*	*1400–1445*	*1430–1461*	*1357–1438*	*1372–1470*	*1411–1489*
	2	5		*1383–1423*	*1400–1441*	*1420–1455*	*1325–1435*	*1347–1465*	*1361–1367* (0.5) *1407–1496* (95.0)
Johnston	n/a	32	late Middle	*1372–1392*	*1399–1465*	*1465–1483*	*1349–1400 (90*.*4) 1410-… (5*.*0)*	*1376–1480*	*1459–1494*
Campbell Farm Late	1	5	Early/Middle	*1395–1420*	*1410–1450*	*1440–1465*	*1381–1432*	*1395–1466*	*1430–1480*
	2	5		*1401–1428*	*1414–1444*	*1431–1457*	*1380–1440*	*1397–1462*	*1420–1478*
Consol Late	1	8	late Middle	*1397–1425*	*1417–1456*	*1446–1471*	*1385–1440*	*1400–1471*	*1438–1485*
	2	8		*1395–1435*	*1417–1455*	*1442–1469*	*1356–1450*	*1388–1476*	*1431–1493*
Household	1	3	Late	*1396–1451*	*1419–1481*	*1448–1502*	*1371–1505*	*1389–1531*	*1423–1553*
	2	3		*1381–1452*	*1410–1485*	*1441–1516*	*1307–1495*	*1351–1547*	*1414–1601*
Kirschner Late	1	6	late Middle	*1428–1455*	*1445–1484*	*1474–1500*	*1415–1465*	*1429–1501*	*1464–1515*
	2	6		*1435–1463*	*1449–1480*	*1465–1494*	*1408–1475*	*1428–1502*	*1457–1518*
Grays Landing 2	1	7	Middle	*1415–1455*	*1437–1487*	*1466–1506*	*1400–1482*	*1415–1515*	*1453–1532*
	2	7		*1397–1457*	*1430–1490*	*1462–1517*	*1349–1490*	*1389–1534*	*1445–1570*
Mon City	1	4	late Middle	*1420–1465*	*1441–1497*	*1470–1515*	*1400–1502*	*1415–1535*	*1453–1551*
	2	4		*1408–1470*	*1435–1500*	*1465–1521*	*1351–1513*	*1392–1554*	*1446–1591*
Brokaw	1	9	Early	*1391–1428*	*1413–1459*	*1445–1476*	*1375–1445*	*1391–1480*	*1430–1495*
	2	9		*1370–1432*	*1405–1463*	*1441–1486*	*1315–1455*	*1360–1495*	*1425–1521*
Squirrel Hill	1	3	Late Middle	*1413–1436*	*1430–1468*	*1461–1486*	*1400–1445*	*1413–1485*	*1452–1498*
	2	3		*1415–1440*	*1429–1469*	*1455–1486*	*1378–1453*	*1402–1499*	*1446–1524*
Throckmorton	1	2	Late	*1465–1527 (33*.*3) 1551–1615 (35*.*0)*	*1495–1554 (31*.*4) 1568–1635 (36*.*9)*	*1516–1574* (30.8) *1594–1664* (37.5)	*1442–1644*	*1460–1670*	*1490–1690*
	2	2		*1464–1524 (33*.*4) 1545–1612 (34*.*8)*	*1493–1555* (30.8) *1561–1634* (37.5)	*1518–1659*	*1426–1645*	*1451–1674*	*1479–1698*

Numbers in parentheses are percentages for bimodal distributions. Model 1 Interval N50,10, Model 2 Interval LnN(ln(50),ln(2)). Agreement indices: Model 1 A_model_ = 121.7, A_overall_ = 118.5; Model 2 A_model_ = 136.2, A_overall_ = 134.0. Johnston site model agreement indices A_model_ = 142.4, A_overall_ = 139.3 (see text for explanation).

### Allegheny Mountains section

Several sites in the Allegheny Mountains section have two rings of houses or evidence for the expansion of existing rings. The inner rings of two-ring villages are generally thought to represent earlier occupations of the sites and the outer rings later occupations [45, 46]. Whether these document the coalescence of separate communities, growth of single communities, or reuse of locations separated in time is unclear. Other sites, such as Petenbrink, have superimposed earlier and later villages. Railroad had a single settlement plan, but some house patterns overlap [80]. The radiocarbon dates from this site fall into two groups so that the overlapping structure patterns appear to represent different occupations of the location rather than simply rebuilding episodes. To understand the occupational histories of the individual sites, Sequential Phase models were run for each of the sites using radiocarbon dates associated with the inner and outer rings as identified by Means [46] at Fort Hill, Gnagey 3, and Peck 1, or any groups of dates representing overlapping occupations as at Peck 2, Petenbrink 1, and Railroad with the normal-distribution Interval constraint. Results are presented in Table 3.

**Table 3:** Date estimates of and intervals between site-specific sequential phases in the Allegheny Mountains section of the lower upper Ohio River basin.

Site Component	Date Estimate (64.3% hpd)	Date Estimate (95.4% hpd)	Interval Between (68.3% hpd)	Interval Between (95.4% hpd)	Agreement Indices	
					model	overall
Fort Hill I	*1233–1271*	*1214–1288*	0–31 (62.3) 63–73 (6.0)	0–90	104.6	104.5
Fort Hill II	*1280–1330* (56.4) *1353–1364* (6.7)	*1275–1396*				
Gnagey 3–1	*1269–1306*	*1250–1325*	0–36	0–70	105.0	103.8
Gnagey 3–2	*1331–1388*	*1310–1415*				
Peck 1 Core	*1294–1335*	*1274–1355*	0–13	0–29	87.5	89.4
Peck 1 Exp. 1	*1347–1387*	*1329–1405*				
			0–15	0–33		
Peck 1 Ex. 2	*1399–1440*	*1381–1461*				
Peck 2–1	*1183–1252*	*1085–1330*	182–257	143–343	87.1	87.9
Peck 2–2	*1447–1525*	*1425–1610*				
Petenbrink 1–1	*1094–1164*	*1037–1180*	26–106	0–145	135.9	134.6
Petenbrink 1–2	*1229–1270*	*1205–1297*				
Railroad I	*1235–1275*	*1215–1294*	13–115	0–131	134.3	135.1
Railroad II	*1315–1417*	*1300–1441*				

The results suggest that occupations at Fort Hill, Gnagey3, and Peck 1 were continuous. That is, the different house rings were occupied simultaneously, or the villages were rearranged/rebuilt without detectable hiatuses in their occupational histories. This accords with community pattern analyses for the sites presented in Means [45, 46]. As a result, each site was included as a single Phase in the multi-Phase models. Peck 2, Petenbrink 1, and Railroad, have occupational histories that indicate hiatuses of several decades to over two centuries. As a result, the different occupations at these sites were included as separate Phases in the multi-Phase models. The results of overlapping subregional models for 12 village Phases are presented in Table 4.

**Table 4:** Start boundaries, Date estimates, and End boundaries for sites/site components in the Allegheny Mountains.

Site/Component	Model	n	Culture-Hist. Time Period	68.3% hpd			95.4% hpd		
				Start Boundary	Date Estimate	End Boundary	Start Boundary	Date Estimate	End Boundary
Petenbrink 1–1	1	7	Early	*1045–1122*	*1068–1150*	*1096–1174*	*1020–1148*	*1036–1180*	*1066–1198*
	2	7		*1036–1121*	*1068–1152*	*1095–1177*	*1015–1155*	*1034–1182*	*1057–1210*
Peck 2–1	1	3	Early	*1123–1189*	*1142–1218*	*1172–1236*	*1020–1074* (16.2) *1102–1203* (79.2)	*1040–1105* (16.0) *1116–1234* (79.4)	*1067–1126* (16.5) *1153–1253* (79.0)
	2	3		*1046–1055* (4.4) *1124–1196* (63.9)	*1064–1068* (1.7) *1141–1219* (66.6)	*1166–1238*	*1026–1206*	*1044–1231*	*1060–1120* (14.8) *1131–1263* (80.6)
Railroad I	1	11	Early	*1214–1244*	*1232–1274*	*1264–1292*	*1197–1258*	*1214–1290*	*1245–1305*
	2	11		*1215–1254*	*1234–1273*	*1255–1290*	*1186–1270*	*1211–1291*	*1238–1305*
Sang Run	1	2	Early	*1191–1254*	*1212–1281*	*1241–1302*	*1145–1275*	*1165–1309*	*1194–1325*
	2	2		*1190–1260*	*1211–1282*	*1235–1302*	*1128–1282*	*1155–1318*	*1185–1345*
Petenbrink 1–2	1	5	Early	*1210–1247*	*1229–1275*	*1258–1295*	*1186–1265*	*1205–1297*	*1237–1313*
	2	5		*1210–1255*	*1228–1275*	*1250–1294*	*1173–1272*	*1197–1301*	*1230–1321*
Fort Hill	1	8	Middle	*1249–1268*	*1264–1299*	*1296–1316*	*1236–1275*	*1250–1314*	*1289–1325*
	2	9		*1252–1279*	*1268–1299*	*1289–1313*	*1226–1286*	*1245–1320*	*1281–1336*
Gnagey 3	1	7	Middle	*1255–1275*	*1271–1310*	*1305–1326*	*1243–1285*	*1256–1326*	*1295–1339*
	2	7		*1244–1280*	*1266–1316*	*1298–1341*	*1210–1293*	*1235–1355*	*1288–1392*
Peck No. 1	1	4	Middle	*1255–1277*	*1271–1310*	*1305–1327*	*1237–1290*	*1251–1330*	*1291–1344*
	2	4		*1246–1284*	*1266–1315*	*1296–1337*	*1199–1297*	*1236–1378*	*1286–1395*
Jonas Field	1	3	Middle	*1248–1302*	*1266–1337*	*1298–1352*	*1240–1376*	*1256–1404*	*1292–1425*
	2	3		*1237–1306* (65.0) *1360–1367* (3.2)	*1268–1336* (62.1) *1369–1383* (6.2)	*1298–1354* (54.3) *1378–1400* (14.0)	*1202–1384*	*1240–1410*	*1287–1433*
Reckner	1	3	Middle	*1293–1338* (52.8) *1371–1391* (15.4)	*1314–1371* (55.2) *1388–1407* (13.1)	*1341–1383* (49.1) *1409–1434* (19.1)	*1283–1400*	*1299–1431*	*1332–1449*
	2	3		*1299–1345* (52.7) *1379–1397* (15.6)	*1316–1365* (47.3) *1386–1414* (21.0)	*1335–1375* (42.1) *1400–1429* (26.1)	*1272–1408*	*1295–1435*	*1325–1456*
Railroad II	1	4	Middle	*1306–1337* (24.9) *1363–1404* (43.4)	*1334–1363* (20.7) *1378–1430* (47.5)	*1354–1363* (20.7) *1378–1430* (47.5)	*1290–1414*	*1308–1448*	*1339–1463*
	2	4		*1309–1350* (35.7) *1370-1406*(32.6)	*1329–1365* (26.8) *1384–1430* (41.4)	*1348–1376* (19.6) *1405–1454* (48.6)	*1284–1421*	*1306–1452*	*1331–1477*
Peck 2–2	1	3	Middle	*1446–1499* (42.1) 1560–1601 (26.1)	*1467–1530* (42.6) 1581–1627 (25.7)	*1496–1530* (42.7) 1609–1649 (26.5)	*1433–1615*	*1453–1644*	*1484–1663*
	2	3		*1445–1507* (46.5) *1567–1606* (21.8)	*1467–1531* (43.0) *1581–1627* (25.3)	*1490–1531* (39.3) *1600–1648* (28.9)	*1426–1621*	*1450–1645*	*1476–1666*

Numbers in parentheses are percentages for bimodal distributions. Model 1 Interval N50,10, Model 2 Interval LnN(ln(50),ln(2)). Agreement indices: Model 1 A_model_ = 146.2, A_overall_ = 144.8 Model 2 A_model_ = 127.0, A_overall_ = 125.9.

As with the Unglaciated Allegany Plateau models, the results of the two Allegheny Mountain models are very similar, with the log-normal distribution Interval constraint model being somewhat less constrained than the normal distribution Interval constraint model. In the normal distribution model several dates had poor agreement indices, which resulted in model agreement indicates only slightly above 60%. Removing these dates from the model, one from Petenbrink 1 early (Beta-104103), and three from Railroad early (Beta-104120, 104131, 104134) resulted in higher agreement indices. As a result, the number of samples used in the two models for these sites differ.

### Site chronological ordering

**Fig 2:**
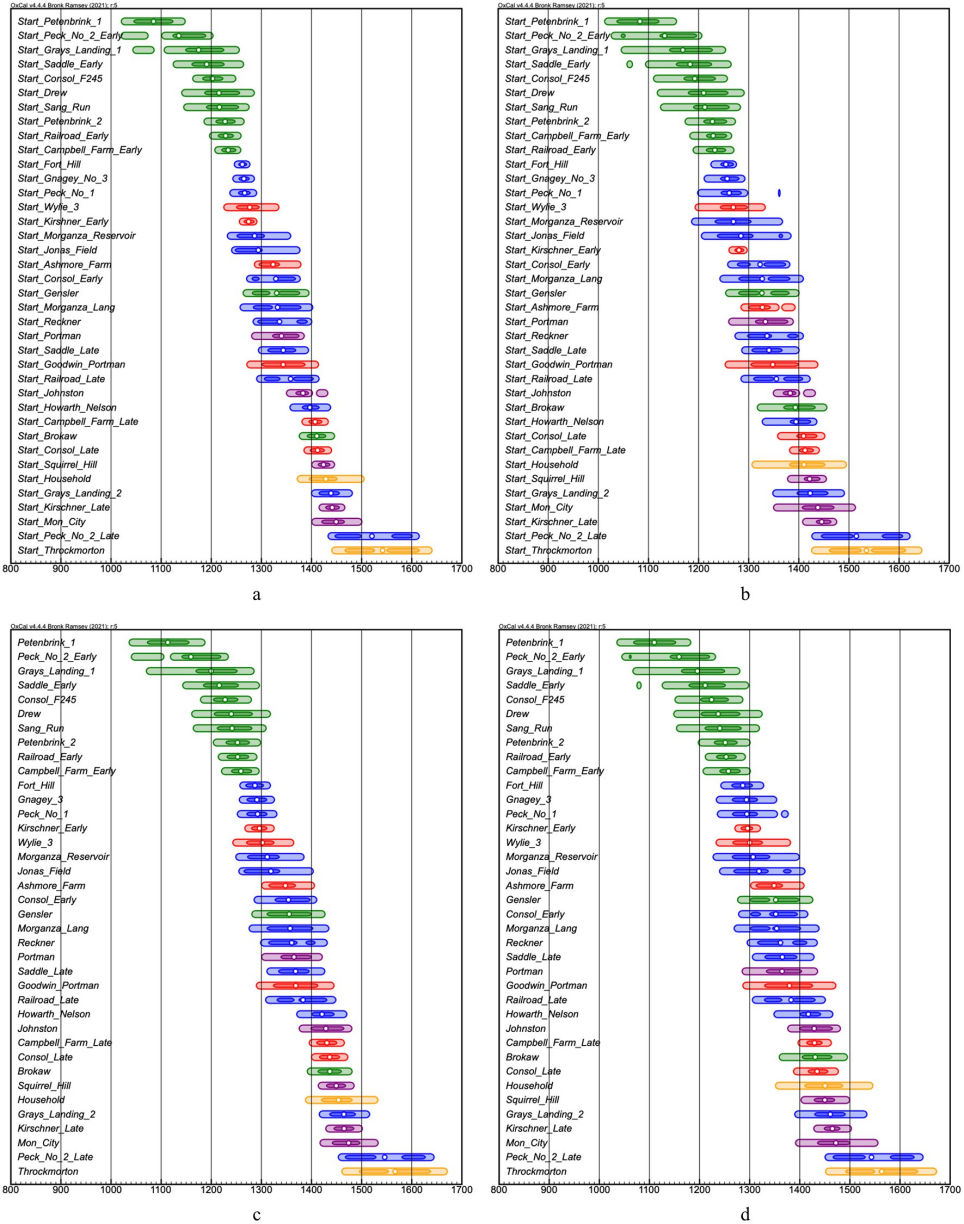
Modeled date ranges for Monongahela tradition sites in the order listed in Table 5: Start Boundaries (a) normal distribution interval constraint and (b) log normal interval constraint; Date estimates (c) normal distribution and (d) log normal distribution. Dark shading is 68.3% hpd, light shading is 94.5% hpd, and white dots are the mean. Green shading = sites assigned to the Early Monongahela period, red = early Middle Monongahela, blue = Middle Monongahela, purple = late Middle Monongahela, and yellow = Late Monongahela.

Site order probability matrices were calculated combining the results of the two subregional models for each interval constraint using the Order command with posterior probability data for Start Boundaries and Date estimates. A summary of the matrices is presented in Table 5 indicating the probability of each site being later in time than the preceding site. Detailed results are provided in S2 Table. The site orders between the different Interval constraints and between the Start Boundaries and Date Estimates are largely in accord with several reversals between two or three sites, the latter of which indicating their probable contemporaneous occupations. The date ranges plotted in Fig 2 are based on the orders in Table 5 along with 68.3% and 94.5% hpd date ranges and median probabilities, shaded according to previous culture-historical time-period assignments.

**Table 5:** Summary of site order probability matrices (see S2 Table).

Start Boundaries				Date Estimate			
Normal Distribution Interval Constraint		Log Normal Distribution Interval Constraint		Normal Distribution Interval Constraint		Log Normal Distribution Interval Constraint	
Site	prob. later	Site	prob. later	Site	prob. later	Site	prob. later
Petenbrink 1	--	Petenbrink 1	--	Petenbrink 1	--	Petenbrink 1	--
Peck No 2–1	0.8125	Peck No 2–1	0.7809	Peck No 2–1	0.7820	Peck No 2–1	0.7861
Grays Landing 1	0.7602	Grays Landing 1	0.7125	Grays Landing 1	0.7425	Grays Landing 1	0.7171
Saddle Early	0.5981	Saddle Early	0.5828	Saddle Early	0.5935	Saddle Early	0.5895
Consol F245	0.6081	Consol F245	0.5790	Consol F245	0.5969	Consol F245	0.6012
Drew	0.6443	Drew	0.6378	Drew	0.6205	Drew	0.6148
Sang Run	0.5017	Sang Run	0.5144	Sang Run	0.5022	Sang Run	0.5230
Petenbrink 2	0.5938	Petenbrink 2	0.6039	Petenbrink 2	0.5861	Petenbrink 2	0.5788
Railroad Early	0.5256	Campbell Farm Early	0.5260	Railroad Early	0.5036	Railroad Early	0.5169
Campbell Farm Early	0.6094	Railroad Early	0.5186	Campbell Farm Early	0.5978	Campbell Farm Early	0.5827
Fort Hill	0.9789	Fort Hill	0.8364	Fort Hill	0.8703	Fort Hill	0.8238
Gnagey No 3	0.6008	Gnagey No 3	0.5853	Gnagey 3	0.5619	Gnagey 3	0.5818
Peck No 1	0.5157	Peck No 1	0.5585	Peck No 1	0.5099	Peck No 1	0.5077
Wylie 3	0.6458	Wylie 3	0.5977	Kirschner Early	0.5722	Kirschner Early	0.5496
Kirshner Early	0.5056	Morganza Reservoir	0.5073	Wylie 3	0.5522	Wylie 3	0.5266
Morganza Res.	0.6053	Jonas Field	0.5881	Morganza Res.	0.5831	Morganza Res.	0.5620
Jonas Field	0.5302	Kirschner Early	0.5110	Jonas Field	0.5323	Jonas Field	0.5662
Ashmore Farm	0.7786	Consol Early	0.8522	Ashmore Farm	0.7503	Ashmore Farm	0.7350
Consol Early	0.5811	Gensler	0.5269	Consol Early	0.5776	Gensler	0.5267
Gensler	0.5166	Ashmore Farm	0.5114	Gensler	0.5091	Consol Early	0.5000
Morganza Lang	0.5105	Morganza Lang	0.5026	Morganza Lang	0.5083	Morganza Lang	0.5109
Reckner	0.5280	Portman	0.5437	Reckner	0.5204	Reckner	0.5498
Portman	0.5567	Reckner	0.5035	Portman	0.5554	Saddle Late	0.5381
Saddle Late	0.5096	Saddle Late	0.5414	Saddle Late	0.5086	Portman	0.5106
Goodwin Portman	0.5074	Goodwin Portman	0.5678	Goodwin Portman	0.5075	Goodwin Portman	0.5960
Railroad Late	0.6046	Railroad Late	0.5395	Railroad Late	0.5962	Railroad Late	0.5186
Johnston	0.6913	Johnston	0.6977	Howarth Nelson	0.7952	Howarth Nelson	0.7666
Howarth Nelson	0.7633	Brokaw	0.6458	Johnston	0.5763	Johnston	0.6101
Campbell Farm Late	0.6841	Howarth Nelson	0.5038	Campbell Farm Late	0.5178	Campbell Farm Late	0.5002
Brokaw	0.5582	Consol Late	0.6815	Consol Late	0.5838	Brokaw	0.5408
Consol Late	0.5273	Campbell Farm Late	0.5261	Brokaw	0.5037	Consol Late	0.5297
Squirrel Hill	0.7572	Household	0.5049	Squirrel Hill	0.6704	Household	0.6355
Household	0.5283	Squirrel Hill	0.6001	Household	0.5367	Squirrel Hill	0.5095
Grays Landing 2	0.6313	Grays Landing 2	0.5280	Grays Landing 2	0.6114	Grays Landing 2	0.6142
Kirschner Late	0.5657	Mon City	0.6195	Kirschner Late	0.5154	Kirschner Late	0.5553
Mon City	0.5711	Kirschner Late	0.5993	Mon City	0.5820	Mon City	0.5509
Peck No 2–2	0.8791	Peck No 2–2	0.8899	Peck No 2–2	0.8590	Peck No 2–2	0.8506
Throckmorton	0.6164	Throckmorton	0.5981	Throckmorton	0.6085	Throckmorton	0.5970

## Discussion

In their recent review of the Monongahela Tradition, Johnson and Means ([38], pp. 349–350) state that: “[w]ithout a stronger notion of a component’s temporal placement, it is difficult to create a developmental framework that would enable the study of variation in Monongahela social organization.” Here we have begun to address this issue with the existing database of radiocarbon dates. The results of the Bayesian analyses, as summarized in Fig 2, show that based on available radiocarbon dates there are distinct groups of sites that are approximately contemporaneous. These occur within and between the traditional Monongahela tradition time period assignments. The importance of this result is that it allows considerations of variation in the decisions made by members of communities on the construction and organization of their built environments [46]. Forcing sites into artificial cultural historical chronological units based primarily on similarity of pottery assemblages and then comparing them across these units can generate a narrative that there is a natural and steady progression in the complexity of village layouts in terms of the number and arrangement of constituent social groups and overall site size. As Means [46] showed for the Allegheny Mountains region and as we show below for the broader Monongahela tradition, this is simply not the case. The layout of Monongahela tradition villages reflected the social institutions that developed to manage the interactions of people who lived in and worked alongside each other on a day-to-day basis [46, 81].

**Fig 3:**
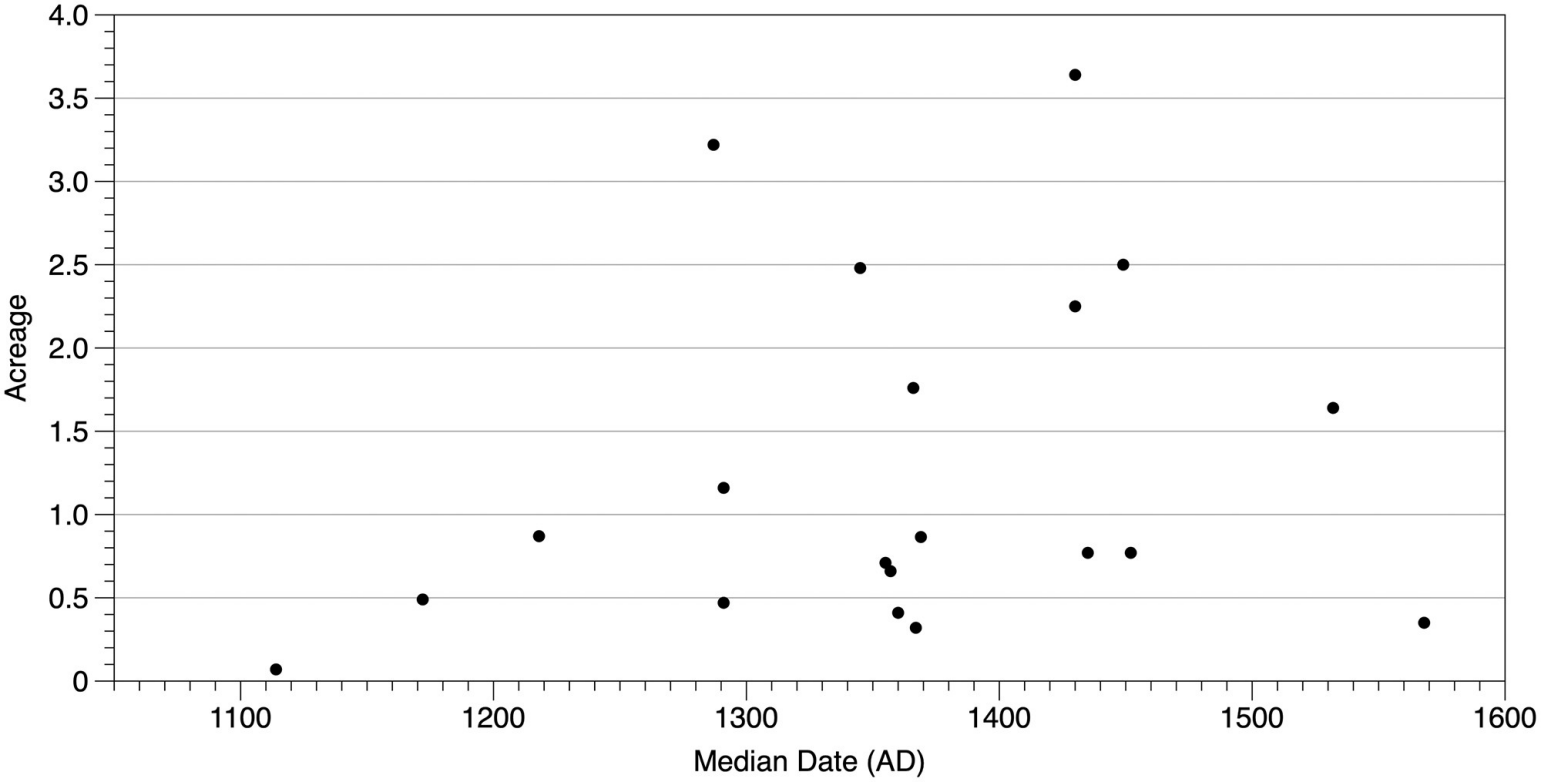
Site acreage by median of the Date estimate.

**Fig 4:**
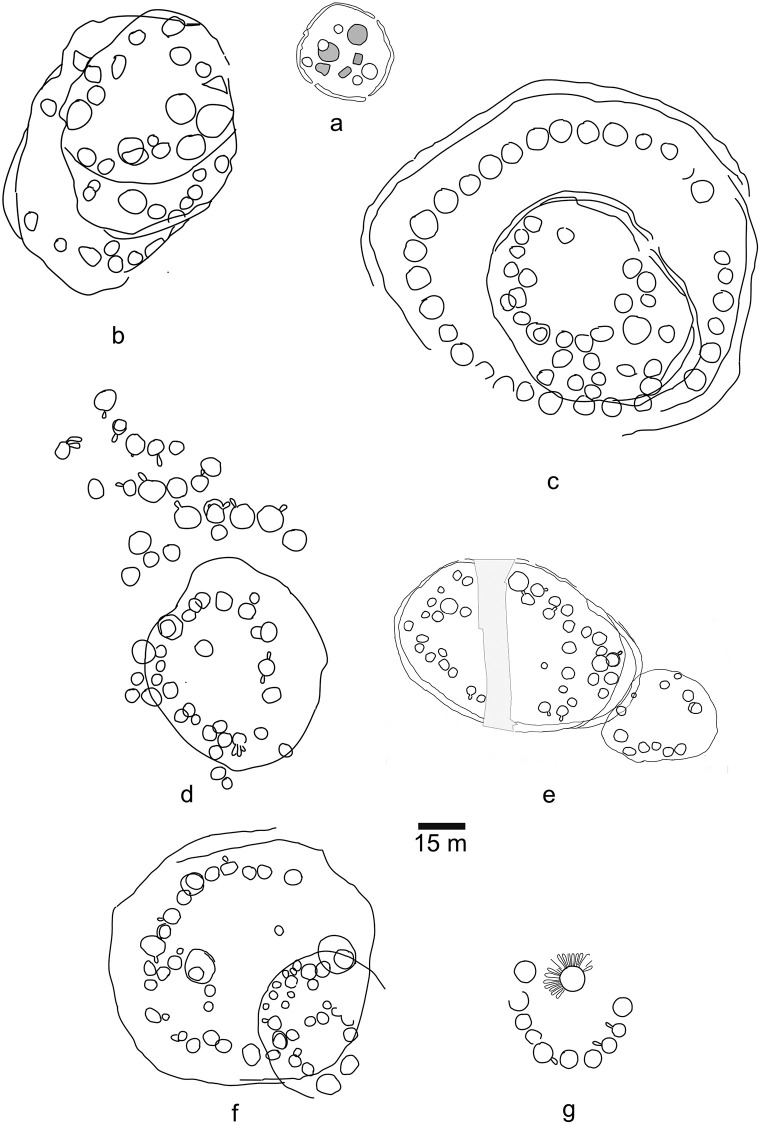
Site plans showing house and palisade outlines as inferred from postmold patterns for village sites discussed in the text: (a) Gnagey No. 3 (shaded structures represent the original site plan), (b) Fort Hill, (c) Peck No. 1, (e) Reckner, (f) Consol (shading represents roadway), (g) Peck No. 2, (f) Throckmorton. Overlapping house patterns represent rebuilding episodes rather than synchronically occupied structures.

Fig 3 is a plot of village acreage by time using the median dates of the estimated Date ranges. As can be seen, there is substantial variation in village size in this small sample, including sites occupied at approximately the same time. A non-parametric Mann-Kendall trend test as implemented in PAST statistical software v. 4.10 [82] on village acreage placed in probable order per Table 5 and S2 Table indicates no statistically significant trend in the data (p = 0.36339) consistent with previous analyses [39, 46]. Rather, the data suggest continuous variation in the manners in which communities constructed their built environments. As noted by Johnson and Means ([38], p. 356), variation in village size “was more directly linked to a village’s constituent social groups and less directly to a village component’s order within the Monongahela temporal sequence.” An examination of completely excavated sites occupied at approximately the same time further demonstrates the variation inherent in Monongahela tradition settlement patterns (Fig 4).

As an example, three completely excavated sites located in the Allegheny Mountains section, Gnagey No. 3, Fort Hill, and Peck No. 1, have 68.3% date estimate ranges that overlap in the ~AD 1270-to-1310-time span. Each of the sites was reconfigured during that time. Gnagey No. 3 began as a small village of 0.29 acres consisting of 5 structures, surrounded by a partial palisade. It was rearranged to encompass 0.47 acres with at least 5 structures. The central plaza was expanded from 79 to 382 square meters. Fort Hill began as a circular, palisaded village encompassing 1.02 acres with 34 structures inside the palisade. It was expanded to encompass 3.22 acres with a second ring of 35 structures. This expansion enlarged the central plaza from 1132 to 6884 square meters assuming the original ring of houses was abandoned and dismantled when the second ring of houses was built [46]. Peck No. 1 was initially a ring-shaped, palisaded village with approximately 15 structures. It was expanded twice, ultimately encompassing 1.16 acres with 38 structures and a 611 square meter plaza. Thus, three villages in close proximity, occupied approximately simultaneously, exhibit very different site plans. While all three were expanded during their occupations, their size and expansions varied considerably.

Peck 1 represented growth by the addition of small social groups on two occasions, perhaps representing discrete families or lineage groups who left another village. Drawing on ethnographic parallels, Means [46] suggested that the palisade was partly dismantled to incorporate each group—perhaps after a waiting period during which they were vetted by the community. Further drawing on ethnographic parallels, it may be that Peck 1 had a particularly charismatic village leader whose presence attracted families from other villages. While Peck 1 became larger because of an increase in village community membership, Gnagey 3 saw no apparent increase in population, but rather was likely reconfigured because the initial occupation failed to adhere to ideal settlement layout models used to plan villages in this region. Gnagey 3 had an extremely small common area at its founding that was dominated by roasting pits with little space between the communal area and the houses that faced this area. When Gnagey 3 was reconfigured and enlarged, the village more closely adhered to an ideal settlement model that characterized most known villages in the region, where houses faced a larger communal area. The initial occupation at Fort Hill was designed according to such an ideal settlement model but what appears to have been internal growth led to impingement that reduced the size of the village’s plaza as well as creating a plaza that was not centrally distributed within the village. Fort Hill’s reconfiguration led to a substantially larger village that better adhered to the ideal settlement model—again—without a significant increase in village population. However well each village community adhered to the ideal settlement model during its occupation, it is clear that variation in size or layout was dependent on internal community dynamics and not to a village’s placement within an arbitrary cultural-historical temporal framework.

A second example is the Reckner site and the early village at the Consol site. The villages are approximately contemporaneous, dating in the fourteenth-century AD. The Reckner site is located in the Allegheny Mountains and the Consol site in the Unglaciated Allegany Plateau. There is only a 53.4% or 55.48% probability based on Date estimates and 53.8 or 58.61% probability based on Start Boundaries that Reckner is later in time than the early village at Consol. Both sites were completely excavated. There are multiple occupations at Reckner, which Means [46] labeled I, II, and III. Occupation I appears to represent a single community encompassing 0.71 acres with 28 structures and a plaza occupying 749 square meters. The palisaded early village at Consol occupied an area of 0.41 acres with a single ring of at least 10 structures and a plaza encompassing 230 square meters. Here too, there is considerable variation in site sizes and plans. Reckner resembles Peck 1 in that growth represented discrete social groups that likely petitioned for inclusion in the village community. While they may have reached the status of community members, this is not archaeologically visible as Reckner’s palisade was not rebuilt to incorporate the new community members. Reckner’s initial village community may have held for stronger adherence to the ideal settlement layout model than the villagers at Peck 1. It would be interesting to expand from the Means [46] focus on the Allegheny Mountains area to see how much variation there is across the Monongahela tradition in terms of variance from this ideal settlement layout model. Here, we can already suggest that adherence to the ideal settlement layout model relative to internal community dynamics seems not to be dependent on time or geography.

Consol’s two components represented discrete occupations with little deviation from the ideal settlement layout model. Given the short apparent period of separation between the two components at Consol, it is likely that the same community constructed both villages, as was argued above for Fort Hill and Gnagey 3. However, because the two village components overlap and intersect one another, the community members would have had to relocate elsewhere at least for a short period after the occupation of the earlier component. The second component was also considerably larger, which would indicate the community grew, although whether internally or from the incorporation of other social groups is not readily evident.

A final example is the late occupation of Peck No. 2 in the Allegheny Mountains and Throckmorton in the Unglaciated Plateau. These villages were occupied close in time during the sixteenth-century AD. The probability that Throckmorton was later than Peck No. 2 of ~61% based on Date estimates and Start Boundaries. The late village at Peck No. 2 occupied an area of 1.64 acres, with palisade, 32 structures, and a 2,243 square meter central plaza. Two of the structures are exceptionally large and are located opposite one another. A small courtyard is suggested by a cluster of eight structures forming a small circle with the larger ring (Means 2006). Throckmorton, on the other hand, occupied an area of only 0.35 acres with 11 structures in a circular pattern, one of which was a petal structure with 18 attached appendages, and a plaza encompassing 354 square meters. No palisade was recorded at the site. Although these two village sites were contemporary, they are assigned to two different temporal units in the Monongahela tradition chronological framework. Scholars that examine village layouts but restrict themselves to working within the confines of this culture-historical chronological framework would be unaware of two different, but contemporaneous strategies adopted by villagers that saw a shift from more autonomous social units within the village to shifts indicating broader community integration (Throckmorton) and co-opting of village space for a smaller social group (the late village at Peck 2). The former strategy seems to have proven more successful as petal houses are noted at other later Monongahela village sites, such as Sony [38].

These sets of approximately contemporaneous sites exhibit substantial differences in original and expanded size and configurations. These reflect decisions made by the sites’ inhabitants to deal with their immediate contingencies. These would include social (e.g., regional political developments) and environmental (e.g., climate change) factors. The results demonstrate how variable were the actions of communities in establishing and modifying their built environments. Limiting radiocarbon assays to chronology building–particularly when used primarily to reinforce extant culture-historical chronological units–ignores their potential for allowing us to address archaeological questions that matter. We can go beyond the pot sherds so enamored by traditionalists mired in the culture history approach and explore how people built village communities that allowed them to address their own local needs.

## Conclusions

Eastern North American archaeology remains committed to early to mid-twentieth-century culture-historical taxonomies. This archaeological practice is detrimental to our understandings of the past. The homogenization of the past into convenient chronological and spatial packages has little if anything to do with the manners in which people lived their lives. Narratives about the past based on culture-historical taxonomies obscure variation in the archaeological record. The recognition and analysis of that variation, on the other hand, adds dimension to narratives of the past, and allows us to better understand how real people lived their lives.

Here we have analyzed a large dataset of radiocarbon dates from sites in the lower upper Ohio River basin dating from ~ AD 1050–1635. The results allowed us to view the past in continuous sequence rather than in arbitrary time periods. They have allowed us to identify village sites that were likely occupied conterminously or at least very close in time. This in turn allowed us to begin to document variation in site settlement patterns that reflect the manners in which communities structured their built environments, expanding on the earlier work of Means [46], which was limited to the Allegheny Mountains section.

The Bayesian analyses are based on the available radiocarbon record, which is less than desirable. As Means [42] documented, the distribution of sites assigned to the Monongahela tradition with available radiocarbon dates is very uneven, both spatially and chronologically. For example, the Johnston site has over 30 radiocarbon dates, many on annual plant products, whereas many of the other sites included in the analysis have only two or a few dates on unidentified charcoal. Other sites in this time span have single dates or have been assigned to one of the time periods based on pottery assemblages. As a result, we are currently unable to place many sites in a chronological framework that is based on Bayesian modeling radiocarbon dates.

Contemporary practice calls for multiple radiocarbon measures on annual plant remains, which do not have potential for built-in ages like wood charcoal. Wood charcoal may be useful through wiggle-match dating [83] or as *termini post quem* [55]. However, these methods have not been applied in the region. Thus, while there is a large database of radiocarbon dates from sites assigned to the Monongahela tradition, many additional dates are needed to fully explore the chronological sequences of sites so that the variation in the manners in which the people responsible for the archaeological record lived their lives. This will enable us to completely abandon legacy archaeological practice and use the archaeological record for the main purpose of archaeology—the elucidation of the lives of real people who existed in contexts vastly different from our own. This needs to be done in combination with other contemporary methods and techniques such as:

- Geophysical surveys of partially excavated village sites, which will help create a larger database of village sizes and settlement patterns (e.g., [84–86]).
- Social network analyses of stylistically varied artifacts such as pottery to better understand how villagers were linked in regional networks of interaction [87, 88].
- Assessments of when European metals and glass beads enter the region, including compositional analysis of cuprous artifacts to determine North American or European source [74, 89]. Based on analyses in Northern Iroquoia [15, 54, 56, 57, 74], the adoption of European items may be different than currently thought.
- Test as a formal hypotheses suppositions linking climate change such the Little Ice Age [62, 64, 65] and/or drought [68] to changes in regional settlement patterns, correlating a robust chronology to contemporary climatic reconstructions (e.g., [90, 91]. For example, did the gradual cooling associated with the Little Ice Age [92] impact subsistence systems?
- Test as a formal hypothesis that the Indigenous groups categorized by archaeologists as the Monongahela tradition actually dispersed by 1635 as a result of depravations by the Seneca [65], possibly combined with an extended period of drought [68]. There is currently no independent chronological basis for the suggestion that the groups dispersed by 1635, prior to European presence in the region, but after possible participation in European-Indigenous trade networks [38].

Johnson and Means [38, p. 382–383] list a wider range of topics that need exploration in the Monongahela tradition region, including more formal analysis of pottery technology rather than simple reliance on pottery types, to gain better understandings of chronological and spatial patterns in how the people represented by this archaeological category lived their lives. As they recognize this can only be done in the context of a robust chronology. The present analyses provide an initial step toward addressing that need consistent with the use of Bayesian analyses of radiocarbon datasets worldwide (e.g., [93–96]).

## Supporting information

S1 Table Radiocarbon dates used in the Bayesian analyses. (XLSX)

S2 Table Site order probability matrices. (XLSX)

S1 Appendix OxCal runfiles for all models. (PDF)
