# Supplementary material for: The Monongahela tradition in “real time”: Bayesian analysis of radiocarbon dates
Source: PLoS One. 2022 Oct 26;17(10):e0276014. doi: 10.1371/journal.pone.0276014 (PMC9605291; doi:10.1371/journal.pone.0276014)
Supplement: S1 Appendix — (PDF) [file pone.0276014.s003.pdf]

Hart JP, Means BK. The "Monongahela Tradition" in "Real Time". S1 Appendix. OxCal runfiles for all models.

Unglaciated Allegheny Plateau Models OxCal Code

Campbell Farm Site Sequential Model

```
Plot()
 {
  Outlier_Model("General",T(5),U(0,4),"t");
  Outlier_Model("Charcoal",Exp(1,-10,0),U(0,3),"t");
  Sequence()
  {
   Boundary("Start Early");
   Phase("Early")
   {
    R_Date("ISGS-A1335",730,20)
    {
     Outlier("General",0.05);
    };
    R_Date("ISGS-A1336",835,20)
    {
     Outlier("General",0.05);
    };
    R_Date("AA-40133",794,38)
    {
     Outlier("General",0.05);
    };
    R_Date("DIC-2240",780,50)
    {
     Outlier("Charcoal",1);
    };
    Interval("Interval Early",N(50,10));
    Date("Date Estimate Early");
   };
   Boundary("End Early");
   Interval("Interval between Campbell Farm Early and Late");
   Boundary("Start Late");
   Phase("Late")
   {
    R_Date("AA-40132",462,38)
    {
     Outlier("General",0.05);
    };
    R_Date("DIC-2241",530,45)
    {
```

```
    Outlier("Charcoal",1);
   };
   R_Date("ISGS-A0773",515,35)
   {
    Outlier("General",0.05);
   };
   R_Date("ISGS-A0774",515,30)
   {
    Outlier("General",0.05);
   };
   //R_Date("M-2204",520,110){Outlier("Charcoal",1);};
   //R_Date("SI-1322",425,105){Outlier("Charcoal",1);};
   //R_Date("SI-1323",1010,95){Outlier("Charcoal",1);};
   R_Date("SI-3672",550,60)
   {
    Outlier("General",0.05);
   };
   Interval("Interval Late",N(50,10));
   Date("Date Estimate Late");
  };
  Boundary("End Late");
 };
};
```

Kirshner Site Sequential Model

```
Plot()
 {
  Outlier_Model("General",T(5),U(0,4),"t");
  Outlier_Model("Charcoal",Exp(1,-10,0),U(0,3),"t");
  Outlier_Model("SSimple",N(0,2),0,"s");
  Sequence()
  {
   Boundary("Start Kirshner Early");
   Phase("Kirschner Early")
   {
    R_Combine("Feature 35")
    {
     Outlier("General",0.05);
     R_Date("GX-21853",630,65)
     {
      Outlier("SSimple",0.05);
     };
     R_Date("ISGS-A0770",715,25)
     {
      Outlier("SSimple",0.05);
     };
```

```
 };
 //R_Date("I-18579",520,80){Outlier("SSimple",0.05);};
 R_Date("ISGS-A0769",710,25)
 {
  Outlier("SSimple",0.05);
 };
 R_Date("ISGS-A1331",675,20)
 {
  Outlier("General",0.05);
 };
 R_Date("ISGS-A1333",620,20)
 {
  Outlier("General",1);
 };
 R_Date("ISGS-A1334",675,20)
 {
  Outlier("General",0.05);
 };
 R_Date("UGa-4690",700,60)
 {
  Outlier("Charcoal",1);
 };
 R_Date("UGa-4691",755,70)
 {
  Outlier("Charcoal",1);
 };
 R_Date("ISGS-A1328",690,20)
 {
  Outlier("General",0.05);
 };
 Interval("Interval Early",N(50,10));
 Date("Date Estimate Early");
};
Boundary("End Kirschner Early");
Interval("Interval between Kirschner Early and Kirschner Late");
Boundary("Start Kirschner Late");
Phase("Kirschner Late")
{
 R_Date("I-18577",490,80)
 {
  Outlier("General",0.05);
 };
 R_Date("ISGS-A1329",410,20)
 {
  Outlier("General",0.05);
 };
```

```
   R_Date("ISGS-A1330",400,20)
   {
    Outlier("General",0.05);
   };
   R_Date("ISGS-A1332",380,20)
   {
    Outlier("General",0.05);
   };
   R_Combine("House 13")
   {
    Outlier("General",0.05);
    R_Date("GX-21854",460,70)
    {
     Outlier("SSimple",0.05);
    };
    R_Date("I-18578",400,80)
    {
     Outlier("SSimple",0.05);
    };
   };
   R_Date("ISGS-A0772",340,30)
   {
    Outlier("General",1);
   };
   Interval("Interval Kirschner Late",N(50,10));
   Date("Date Estimate Kirschner Late");
  };
  Boundary("End Kirshner Late");
 };
};
```

Consol Site Sequential Model

```
Plot()
 {
 Outlier_Model("General",T(5),U(0,4),"t");
 Outlier_Model("Charcoal",Exp(1,-10,0),U(0,3),"t");
 Outlier_Model("SSimple",N(0,2),0,"s");
 Sequence()
 {
  Boundary("Start Consol F245");
  Phase("Consol F245")
  {
   //R_Date("ISGS-A6006,F245 charcoal",940,60)
   //{
    //Outlier("Charcoal",0.05);
   //};
   R_Date("Beta-240471,F245 charcoal",900,60)
```

```
 {
  Outlier("Charcoal",1);
 };
 R_Date("ISGS-A1337,F245 maize",795,20)
 {
  Outlier("General",0.05);
 };
 Interval("Interval Consol F245",N(50,10));
 Date("Date Estimate Consol F245");
};
Boundary("End Consol F245");
Interval("Interval between Consol F245 and Consol Early");
Boundary("Start Consol Early");
Phase("Consol Early")
{
 R_Date("ISGS-A1338,F460,maize",680,20)
 {
  Outlier("General",0.05);
 };
 R_Date("Beta-2040465,F468/B24,charcoal",570,60)
 {
  Outlier("Charcoal",1);
 };
 R_Date("ISGS-A4070,F1228,maize",610,20)
 {
  Outlier("General",0.05);
 };
 //R_Date("Beta-299410,F956,charcoal",540,30)
 //{
  //Outlier("Charcoal",1);
 //};
 Interval("Interval Consol Early",N(50,10));
 Date("Date Estimate Consol Early");
};
Boundary("End Consol Early");
Interval("Interval between Consol Early and Consol Late");
Boundary("Start Consol Late");
Phase("Consol Late")
{
 R_Date("Beta-164643 F49/B6",640,70)
 {
  Outlier("Charcoal",1);
 };
 R_Date("Beta-172012,F36/B4,charcoal",550,60)
 {
  Outlier("Charcoal",1);
```

```
   };
   R_Date("Beta-189627,F175/B8,charcoal",550,50)
   {
    Outlier("Charcoal",1);
   };
   R_Date("Beta-149289,F3/B1",540,50)
   {
    Outlier("Charcoal",1);
   };
   R_Date("Beta-172013,F130,charcoal",490,50)
   {
    Outlier("Charcoal",1);
   };
   R_Date("Beta-189128,F190,charcoal",470,70)
   {
    Outlier("Charcoal",1);
   };
   R_Combine("F266,House7Gh")
   {
    Outlier("General",0.05);
    R_Date("ISGS-A0767,bean",450,30)
    {
     Outlier("SSimple",0.05);
    };
    R_Date("ISGS-A0766,maize",405,30)
    {
     Outlier("SSimple",0.05);
    };
   };
   Interval("Interval Consol Late",N(50,10));
   Date("Date Estimate Consol Late");
  };
  Boundary("End Consol Late");
 };
};
```

Saddle Site Sequential Model

```
Plot()
 {
  Outlier_Model("General",T(5),U(0,4),"t");
  Outlier_Model("Charcoal",Exp(1,-10,0),U(0,3),"t");
  Outlier_Model("SSimple",N(0,2),0,"s");
  Sequence()
  {
   Boundary("Start Saddle Early");
   Phase("Saddle Early")
```

```
{
 R_Date("Beta-46466",920,60)
 {
  Outlier("Charcoal",1);
 };
 R_Date("Beta-46469",900,60)
 {
  Outlier("Charcoal",1);
 };
 R_Date("Beta-48979",830,60)
 {
  Outlier("Charcoal",1);
 };
 R_Date("Beta-48980",780,60)
 {
  Outlier("Charcoal",1);
 };
 Interval("Interval Saddle Early",N(50,10));
 Date("Date Estimate Saddle Early");
};
Boundary("End Early");
Interval("Interval Between Saddle Early and Late");
Boundary("Start Saddle Late");
Phase("Saddle Late")
{
 R_Combine("Feature 35")
 {
  Outlier("General",0.05);
  R_Date("AA-38457",675,33)
  {
   Outlier("SSimple",1);
  };
  R_Date("AA-38458",605,34)
  {
   Outlier("SSimple",1);
  };
 };
 R_Date("Beta-46465",550,50)
 {
  Outlier("Charcoal",1);
 };
 R_Date("Beta-46467",580,70)
 {
  Outlier("Charcoal",1);
 };
 R_Date("Beta-46468",590,50)
```

```
    {
     Outlier("Charcoal",1);
    };
    R_Date("Beta-46470",590,70)
    {
     Outlier("Charcoal",1);
    };
    Interval("Interval Saddle Late",N(50,10));
    Date("Date Estimate Saddle Late")
    {
     color="red";
    };
   };
   Boundary("End Saddle Late");
  };
 };
```

Regional Model 1 for Unglaciated Allegheny Plateau sites

```
Plot()
 {
  Outlier_Model("General",T(5),U(0,4),"t");
  Outlier_Model("Charcoal",Exp(1,-10,0),U(0,3),"t");
  Outlier_Model("SSimple",N(0,2),0,"s");
  Sequence()
  {
   Boundary("Start Grays Landing 1");
   Phase ("Grays Landing 1")
   {
    //R_Date("Beta-23591",560,70){Outlier("Charcoal",1);};
    R_Date("Beta-31810",830,70)
    {
     Outlier("Charcoal",1);
    };
    R_Date("Beta-31811",830,70)
    {
     Outlier("Charcoal",1);
    };
    // R_Date("Beta-31812",450,70){Outlier("Charcoal",1);};
    R_Date("Beta-31813",970,80)
    {
     Outlier("Charcoal",1);
    };
    R_Date("Beta-31814",860,60)
    {
     Outlier("Charcoal",1);
```

```
  };
  Interval("Interval Grays Landing 1",N(50,10));
  Date("Grays Landing 1")
  {
   color="green";
  };
 };
 Boundary("End Grays Landing 1");
};
Sequence()
{
 Boundary("Start Consol F245");
 Phase("Consol F245")
 {
  R_Date("ISGS-A6006,F245 charcoal",940,60)
  {
   Outlier("Charcoal",1);
  };
  R_Date("Beta-240471,F245 charcoal",900,60)
  {
   Outlier("Charcoal",1);
  };
  R_Date("ISGS-A1337,F245 maize",795,20)
  {
   Outlier("General",0.05);
  };
  Interval("Interval Consol F245",N(50,10));
  Date("Consol F245")
  {
   color="green";
  };
 };
 Boundary("End Consol F245");
};
Sequence()
{
 Boundary("Start Saddle Early");
 Phase("Saddle Early")
 {
  R_Date("Beta-46466",920,60)
  {
   Outlier("Charcoal",1);
  };
  R_Date("Beta-46469",900,60)
  {
   Outlier("Charcoal",1);
```

```
  };
  R_Date("Beta-48979",830,60)
  {
   Outlier("Charcoal",1);
  };
  R_Date("Beta-48980",780,60)
  {
   Outlier("Charcoal",1);
  };
  Interval("Interval Saddle Early",N(50,10));
  Date("Date Estimate Saddle Early");
 };
 Boundary("End Early");
};
Sequence()
{
 Boundary("Start Drew");
 Phase ("Drew")
 {
  R_Date("Beta-31450",810,60)
  {
   Outlier("Charcoal",1);
  };
  R_Date("Beta-31451",830,50)
  {
   Outlier("Charcoal",1);
  };
  //R_Date("M-2198",830,100){Outlier("Charcoal",1);};
  Interval("Interval Drew",N(50,10));
  Date("Drew")
  {
   color="green";
  };
 };
 Boundary("End Drew");
};
Sequence()
{
 Boundary("Start Campbell Farm Early");
 Phase ("Campbell Farm Early")
 {
  R_Date("ISGS-A1335",730,20)
  {
   Outlier("General",0.05);
  };
  R_Date("ISGS-A1336",835,20)
```

```
  {
   Outlier("General",0.05);
  };
  R_Date("AA-40133",794,38)
  {
   Outlier("General",0.05);
  };
  R_Date("DIC-2240",780,50)
  {
   Outlier("General",0.05);
  };
  Interval("Interval Campbell Farm Early",N(50,10));
  Date("Campbell Farm Early")
  {
   color="green";
  };
 };
 Boundary("End Campbell Farm Early");
};
Sequence()
{
 Boundary("Start Kirshner Early");
 Phase("Kirschner Early")
 {
  R_Combine("Feature 35")
  {
   Outlier("General",0.05);
   R_Date("GX-21853",630,65)
   {
    Outlier("SSimple",0.05);
   };
   R_Date("ISGS-A0770",715,25)
   {
    Outlier("SSimple",0.05);
   };
  };
  //R_Date("I-18579",520,80){Outlier("General",0.05);};
  R_Date("ISGS-A0769",710,25)
  {
   Outlier("General",0.05);
  };
  R_Date("ISGS-A1331",675,20)
  {
   Outlier("General",0.05);
  };
  R_Date("ISGS-A1333",620,20)
```

```
  {
   Outlier("General",0.05);
  };
  R_Date("ISGS-A1334",675,20)
  {
   Outlier("General",0.05);
  };
  R_Date("UGa-4690",700,60)
  {
   Outlier("Charcoal",1);
  };
  R_Date("UGa-4691",755,70)
  {
   Outlier("Charcoal",1);
  };
  R_Date("ISGS-A1328",690,20)
  {
   Outlier("General",0.05);
  };
  Interval("Interval Kirschner Early",N(50,10));
  Date("Kirschner Early")
  {
   color="green";
  };
 };
 Boundary("End Kirschner Early");
};
Sequence()
{
 Boundary("Start Wylie 3");
 Phase ("Wylie 3")
 {
  R_Date("Beta-33183",780,60)
  {
   Outlier("Charcoal",1);
  };
  R_Date("Beta-33184",780,60)
  {
   Outlier("Charcoal",1);
  };
  //R_Date("Beta-33188",450,70){Outlier("Charcoal",1);};
  R_Date("Beta-40613",650,80)
  {
   Outlier("Charcoal",1);
  };
  R_Date("Beta-47106",720,80)
```

```
  {
   Outlier("Charcoal",1);
  };
  //R_Date("Beta-48223",400,70){Outlier("Charcoal",1);};
  R_Date("I-16767",600,80)
  {
   Outlier("Charcoal",1);
  };
  Interval("Interval Wylie 3",N(50,10));
  Date("Wylie 3")
  {
   color="green";
  };
 };
 Boundary("End Wylie 3");
};
Sequence()
{
 Boundary("Start Morganza Reservoir");
 Phase("Morganza Reservoir")
 {
  R_Date("Beta-28096",820,50)
  {
   Outlier("Charcoal",1);
  };
  R_Date("Beta-28095",690,80)
  {
   Outlier("Charcoal",1);
  };
  R_Date("Beta-28094",600,60)
  {
   Outlier("Charcoal",1);
  };
  Interval("Interval Morganza Reservoir",N(50,10));
  Date("Morganza Reservoir");
 };
 Boundary("End Morganza Reservoir");
};
Sequence()
{
 Boundary("Start Ashmore Farm");
 Phase("Ashmore Farm")
 {
  R_Date("ISGS-A-10696",525,45)
  {
   Outlier("General",0.05);
```

```
  };
  R_Date("ISGS-A-10697",570,80)
  {
   Outlier("General",0.05);
  };
  R_Date("ISGS-A10699",600,40)
  {
   Outlier("General",0.05);
  };
  R_Date("ISGS-A10700",575,40)
  {
   Outlier("General",0.05);
  };
  R_Date("ISGS-A10701",590,40)
  {
   Outlier("General",0.05);
  };
  //R_Date("ISGS-A10705",705,55){Outlier("General",0.05);};
  R_Date("ISGS-A10706",640,40)
  {
   Outlier("General",0.05);
  };
  R_Date("ISGS-A10707",630,50)
  {
   Outlier("General",0.05);
  };
  R_Date("ISGS-A10708",630,60)
  {
   Outlier("General",0.05);
  };
  //R_Date("Beta-48175",540,50){Outlier("General",0.05);};
  Interval("Interval Ashmore Farm",N(50,10));
  Date("Ashmore Farm")
  {
   color="blue";
  };
 };
 Boundary("End Ashmore Farm");
};
Sequence()
{
 Boundary("Start Consol Early");
 Phase("Consol Early")
 {
  R_Date("ISGS-A1338,F460,maize",680,20)
  {
```

```
   Outlier("General",0.05);
  };
  R_Date("Beta-2040465,F468/B24,charcoal",570,60)
  {
   Outlier("Charcoal",1);
  };
  R_Date("ISGS-A4070,F1228,maize",610,20)
  {
   Outlier("General",0.05);
  };
  //R_Date("Beta-299410,F956,charcoal",540,30){Outlier("Charcoal",1);};
  Interval("Interval Consol Early",N(50,10));
  Date("Consol Early")
  {
   color="green";
  };
 };
 Boundary("End Consol Early");
};
Sequence()
{
 Boundary("Start Gensler");
 Phase("Gensler")
 {
  R_Date("Dic-2599",630,65)
  {
   Outlier("Charcoal",1);
  };
  R_Date("Dic-2600",650,55)
  {
   Outlier("Charcoal",1);
  };
  R_Date("Dic-2601",690,50)
  {
   Outlier("Charcoal",1);
  };
  Interval("Interval Gensler",N(50,10));
  Date("Gensler")
  {
   color="green";
  };
 };
 Boundary("End Gensler");
};
Sequence()
{
```

```
 Boundary("Start Portman");
 Phase("Portman")
 {
  R_Date("AA-38456",680,35)
  {
   Outlier("General",0.05);
  };
  R_Date("Beta-57298",560,50)
  {
   Outlier("Charcoal",1);
  };
  R_Date("UGa-1643",530,90)
  {
   Outlier("Charcoal",1);
  };
  Interval("Interval Portman",N(50,10));
  Date("Portman")
  {
   color="red";
  };
 };
 Boundary("End Portman");
};
Sequence()
{
 Boundary("Start Goodwin-Portman");
 Phase("Goodwin-Portman")
 {
  R_Date("Beta-153248",510,60)
  {
   Outlier("Charcoal",1);
  };
  R_Date("M-2203",740,100)
  {
   Outlier("Charcoal",1);
  };
  R_Date("Beta-153000",680,60)
  {
   Outlier("Charcoal",1);
  };
  Interval("Interval Goodwin-Portman",N(50,10));
  Date("Goodwin-Portman")
  {
   color="red";
  };
 };
```

```
 Boundary("End Goodwin-Portman");
};
Sequence()
{
 Boundary("Start Saddle Late");
 Phase("Saddle Late")
 {
  R_Combine("Feature 35")
  {
   Outlier("General",0.05);
   R_Date("AA-38457",675,33)
   {
    Outlier("SSimple",1);
   };
   R_Date("AA-38458",605,34)
   {
    Outlier("SSimple",1);
   };
  };
  R_Date("Beta-46465",550,50)
  {
   Outlier("Charcoal",1);
  };
  R_Date("Beta-46467",580,70)
  {
   Outlier("Charcoal",1);
  };
  R_Date("Beta-46468",590,50)
  {
   Outlier("Charcoal",1);
  };
  R_Date("Beta-46470",590,70)
  {
   Outlier("Charcoal",1);
  };
  Interval("Interval Saddle Late",N(50,10));
  Date("Date Estimate Saddle Late")
  {
   color="red";
  };
 };
 Boundary("End Saddle Late");
};
Sequence()
{
 Boundary("Start Morganza Lang");
```

```
 Phase("Morganza Lang")
 {
  R_Date("Beta-19161",650,70)
  {
   Outlier("Charcoal",1);
  };
  //R_Date("Beta-19162",490,110){Outlier("Charcoal",1);};
  //R_Date("Beta-245150",410,40){Outlier("Charcoal",1);};
  R_Date("Beta-245152",660,40)
  {
   Outlier("Charcoal",1);
  };
  Interval("Interval Morganza Lang",N(50,10));
  Date("Date Estimate Morganza Lang")
  {
   color="red";
  };
 };
 Boundary("End Morganza Lang");
};
Sequence()
{
 Boundary("Start Howarth-Nelson");
 Phase("Howarth-Nelson")
 {
  R_Date("PITT-38",530,35)
  {
   Outlier("Charcoal",1);
  };
  R_Date("PITT-37",530,35)
  {
   Outlier("Charcoal",1);
  };
  R_Date("PITT-36",580,40)
  {
   Outlier("Charcoal",1);
  };
  R_Date("PITT-39",505,35)
  {
   Outlier("Charcoal",1);
  };
  R_Date("PITT-274",625,50)
  {
   Outlier("Charcoal",1);
  };
  Interval("Interval Howarth-Nelson",N(50,10));
```

```
  Date("Howarth-Nelson")
  {
   color="red";
  };
 };
 Boundary("End Howarth-Nelson");
};
Sequence()
{
 Boundary("Start Campbell Farm Late");
 Phase("Campbell Farm Late")
 {
  R_Date("AA-40132",462,38)
  {
   Outlier("General",0.05);
  };
  R_Date("DIC-2241",530,45)
  {
   Outlier("Charcoal",1);
  };
  R_Date("ISGS-A0773",515,35)
  {
   Outlier("Charcoal",1);
  };
  R_Date("ISGS-A0774",515,30)
  {
   Outlier("Charcoal",1);
  };
  //R_Date("M-2204",520,110){Outlier("Charcoal",1);};
  //R_Date("SI-1322",425,105){Outlier("Charcoal",1);};
  //R_Date("SI-1323",1010,95){Outlier("Charcoal",1);};
  R_Date("SI-3672",550,60)
  {
   Outlier("General",0.05);
  };
  Date("Campbell Farm Late")
  {
   color="blue";
  };
  Interval("Interval  Campbell Farm Late",N(50,10));
 };
 Boundary("End  Campbell Farm Late");
};
Sequence()
{
 Boundary("Start Consol Late");
```

```
 Phase("Consol Late")
 {
  R_Date("Beta-164643 F49/B6",640,70)
  {
   Outlier("Charcoal",1);
  };
  R_Date("Beta-172012,F36/B4,charcoal",550,60)
  {
   Outlier("Charcoal",1);
  };
  R_Date("Beta-189627,F175/B8,charcoal",550,50)
  {
   Outlier("Charcoal",1);
  };
  R_Date("Beta-149289,F3/B1",540,50)
  {
   Outlier("Charcoal",1);
  };
  R_Date("Beta-172013,F130,charcoal",490,50)
  {
   Outlier("Charcoal",1);
  };
  R_Date("Beta-189128,F190,charcoal",470,70)
  {
   Outlier("Charcoal",1);
  };
  R_Combine("F266,House7Gh")
  {
   Outlier("General",0.05);
   R_Date("ISGS-A0767,bean",450,30)
   {
    Outlier("SSimple",0.05);
   };
   R_Date("ISGS-A0766,maize",405,30)
   {
    Outlier("SSimple",0.05);
   };
  };
  Interval("Interval Consol Late",N(50,10));
  Date("Consol Late")
  {
   color="red";
  };
 };
 Boundary("End Consol Late");
};
```

```
Sequence()
{
 Boundary("Start Household");
 Phase("Household")
 {
  R_Date("I-16747",490,80)
  {
   Outlier("Charcoal",1);
  };
  R_Date("UGa-3453",325,80)
  {
   Outlier("Charcoal",1);
  };
  R_Date("Beta-249013",570,40)
  {
   Outlier("Charcoal",1);
  };
  Interval("Interval Household",N(50,10));
  Date("Household")
  {
   color="orange";
  };
 };
 Boundary("End Household");
};
Sequence()
{
 Boundary("Start Kirschner Late");
 Phase("Kirschner Late")
 {
  R_Date("I-18577",490,80)
  {
   Outlier("General",0.05);
  };
  R_Date("ISGS-A1329",410,20)
  {
   Outlier("General",0.05);
  };
  R_Date("ISGS-A1330",400,20)
  {
   Outlier("General",0.05);
  };
  R_Date("ISGS-A1332",380,20)
  {
   Outlier("General",0.05);
  };
```

```
  R_Combine("House 13")
  {
   Outlier("General",0.05);
   R_Date("GX-21854",460,70)
   {
    Outlier("SSimple",0.05);
   };
   R_Date("I-18578",400,80)
   {
    Outlier("SSimple",0.05);
   };
  };
  //R_Date("ISGS-A0772",340,30){Outlier("General",0.05);};
  Interval("Interval Kirschner Late",N(50,10));
  Date("Kirschner Late")
  {
   color="red";
  };
 };
 Boundary("End Kirschner Late");
};
Sequence()
{
 Boundary("Start Grays Landing 2");
 Phase("Grays Landing 2")
 {
  R_Date("UGa-5954",397,79)
  {
   Outlier("Charcoal",1);
  };
  R_Date("UGa-5955",559,50)
  {
   Outlier("Charcoal",1);
  };
  R_Date("UGa-5958",362,48)
  {
   Outlier("Charcoal",1);
  };
  R_Date("UGa-5959",299,99)
  {
   Outlier("Charcoal",1);
  };
  //R_Date("UGa-5960",490,103){Outlier("Charcoal",1);};
  R_Date("UGa-5962",460,88)
  {
   Outlier("Charcoal",1);
```

```
  };
  //R_Date("UGa-5964",254,58){Outlier("Charcoal",1);};
  R_Date("UGa-5965",663,91)
  {
   Outlier("Charcoal",1);
  };
  //R_Date("UGa-5968",600,126){Outlier("Charcoal",1);};
  R_Date("UGa-5969",467,70)
  {
   Outlier("Charcoal",1);
  };
  //R_Date("UGa-5970",467,100){Outlier("Charcoal",1);};
  //R_Date("UGa-5972",427,109){Outlier("Charcoal",1);};
  Interval("Interval Grays Landing 2",N(50,10));
  Date("Grays Landing 2")
  {
   color="red";
  };
 };
 Boundary("End Grays Landing 2");
};
Sequence()
{
 Boundary("Start Mon City");
 Phase("Mon City")
 {
  R_Date("Beta-15280",330,50)
  {
   Outlier("Charcoal",1);
  };
  R_Date("Beta-15819",420,60)
  {
   Outlier("Charcoal",1);
  };
  R_Date("Beta-15821",530,70)
  {
   Outlier("Charcoal",1);
  };
  R_Date("Beta-15822",530,70)
  {
   Outlier("Charcoal",1);
  };
  Interval("Interval Mon City",N(50,10));
  Date("Mon City")
  {
   color="red";
```

```
  };
 };
 Boundary("End Mon City");
};
Sequence()
{
 Boundary("Start Brokaw");
 Phase("Brokaw")
 {
  //R_Date("Dic-391",360,110){Outlier("Charcoal",1);};
  R_Date("Dic-392",740,55)
  {
   Outlier("Charcoal",1);
  };
  R_Date("N-3481",505,75)
  {
   Outlier("Charcoal",1);
  };
  R_Date("N-3482",525,75)
  {
   Outlier("Charcoal",1);
  };
  R_Date("N-3483",460,55)
  {
   Outlier("Charcoal",1);
  };
  //R_Date("TEM-167",530,120){Outlier("Charcoal",1);};
  //R_Date("TEM-168",720,100){Outlier("Charcoal",1);};
  R_Date("TEM-169",620,70)
  {
   Outlier("Charcoal",1);
  };
  R_Date("TEM-185",590,80)
  {
   Outlier("Charcoal",1);
  };
  R_Date("UGa-3429",405,65)
  {
   Outlier("Charcoal",1);
  };
  R_Date("UGa-3430",520,65)
  {
   Outlier("Charcoal",1);
  };
  R_Date("UGa-3943",390,70)
  {
```

```
   Outlier("Charcoal",1);
  };
  Interval("Interval Brokaw",N(50,10));
  Date("Date Estimate Brokaw")
  {
   color="red";
  };
 };
 Boundary("End Brokaw");
};
Sequence()
{
 Boundary("Start Squirrel Hill");
 Phase("Squirrel Hill")
 {
  R_Date("ISGS-A2777",495,25)
  {
   Outlier("General",0.05);
  };
  R_Date("ISGS-A2776",410,20)
  {
   Outlier("General",0.05);
  };
  R_Date("Beta-339435",400,30)
  {
   Outlier("General",0.05);
  };
  Interval("Interval Squirrel Hill",N(50,10));
  Date("Date Estimate Squirrel Hill")
  {
   color="red";
  };
 };
 Boundary("End Squirrel Hill");
};
Sequence()
{
 Boundary("Start Throckmorton");
 Phase("Throckmorton")
 {
  R_Date("Dic-2602",400,50)
  {
   Outlier("Charcoal",1);
  };
  R_Date("Dic-2604",300,60)
  {
```

```
    Outlier("Charcoal",1);
   };
   Interval("Interval Throckmorton",N(50,10));
   Date("Throckmorton")
   {
    color="orange";
   };
  };
  Boundary("End Throckmorton");
 };
 Order("Order Sites");
};
```

Regional Model 2 for Unglaciated Allegheny Plateau sites

```
Plot()
 {
 Outlier_Model("General",T(5),U(0,4),"t");
 Outlier_Model("Charcoal",Exp(1,-10,0),U(0,3),"t");
 Outlier_Model("SSimple",N(0,2),0,"s");
 Sequence()
 {
  Boundary("Start Grays Landing 1");
  Phase ("Grays Landing 1")
  {
   //R_Date("Beta-23591",560,70){Outlier("Charcoal",1);};
   R_Date("Beta-31810",830,70)
   {
    Outlier("Charcoal",1);
   };
   R_Date("Beta-31811",830,70)
   {
    Outlier("Charcoal",1);
   };
   // R_Date("Beta-31812",450,70){Outlier("Charcoal",1);};
   R_Date("Beta-31813",970,80)
   {
    Outlier("Charcoal",1);
   };
   R_Date("Beta-31814",860,60)
   {
    Outlier("Charcoal",1);
   };
   Interval("Interval Grays Landing 1",LnN(ln(50),ln(2)));
   Date("Grays Landing 1")
   {
```

```
   color="green";
  };
 };
 Boundary("End Grays Landing 1");
};
Sequence()
{
 Boundary("Start Consol F245");
 Phase("Consol F245")
 {
  R_Date("ISGS-A6006,F245 charcoal",940,60)
  {
   Outlier("Charcoal",1);
  };
  R_Date("Beta-240471,F245 charcoal",900,60)
  {
   Outlier("Charcoal",1);
  };
  R_Date("ISGS-A1337,F245 maize",795,20)
  {
   Outlier("General",0.05);
  };
  Interval("Interval Consol F245",LnN(ln(50),ln(2)));
  Date("Consol F245")
  {
   color="green";
  };
 };
 Boundary("End Consol F245");
};
Sequence()
{
 Boundary("Start Saddle Early");
 Phase("Saddle Early")
 {
  R_Date("Beta-46466",920,60)
  {
   Outlier("Charcoal",1);
  };
  R_Date("Beta-46469",900,60)
  {
   Outlier("Charcoal",1);
  };
  R_Date("Beta-48979",830,60)
  {
   Outlier("Charcoal",1);
```

```
  };
  R_Date("Beta-48980",780,60)
  {
   Outlier("Charcoal",1);
  };
  Interval("Interval Saddle Early",LnN(ln(50),ln(2)));
  Date("Date Estimate Saddle Early");
 };
 Boundary("End Early");
};
Sequence()
{
 Boundary("Start Drew");
 Phase ("Drew")
 {
  R_Date("Beta-31450",810,60)
  {
   Outlier("Charcoal",1);
  };
  R_Date("Beta-31451",830,50)
  {
   Outlier("Charcoal",1);
  };
  //R_Date("M-2198",830,100){Outlier("Charcoal",1);};
  Interval("Interval Drew",LnN(ln(50),ln(2)));
  Date("Drew")
  {
   color="green";
  };
 };
 Boundary("End Drew");
};
Sequence()
{
 Boundary("Start Campbell Farm Early");
 Phase ("Campbell Farm Early")
 {
  R_Date("ISGS-A1335",730,20)
  {
   Outlier("General",0.05);
  };
  R_Date("ISGS-A1336",835,20)
  {
   Outlier("General",0.05);
  };
  R_Date("AA-40133",794,38)
```

```
   {
    Outlier("General",0.05);
   };
   R_Date("DIC-2240",780,50)
   {
    Outlier("General",0.05);
   };
   Interval("Interval Campbell Farm Early",LnN(ln(50),ln(2)));
   Date("Campbell Farm Early")
   {
    color="green";
   };
  };
  Boundary("End Campbell Farm Early");
 };
 Sequence()
 {
  Boundary("Start Kirshner Early");
  Phase("Kirschner Early")
  {
   R_Combine("Feature 35")
   {
    Outlier("General",0.05);
    R_Date("GX-21853",630,65)
    {
     Outlier("SSimple",0.05);
    };
    R_Date("ISGS-A0770",715,25)
    {
     Outlier("SSimple",0.05);
    };
   };
   //R_Date("I-18579",520,80){Outlier("General",0.05);};
   R_Date("ISGS-A0769",710,25)
   {
    Outlier("General",0.05);
   };
   R_Date("ISGS-A1331",675,20)
   {
    Outlier("General",0.05);
   };
   R_Date("ISGS-A1333",620,20)
   {
    Outlier("General",0.05);
   };
   R_Date("ISGS-A1334",675,20)
```

```
   {
    Outlier("General",0.05);
   };
   R_Date("UGa-4690",700,60)
   {
    Outlier("Charcoal",1);
   };
   R_Date("UGa-4691",755,70)
   {
    Outlier("Charcoal",1);
   };
   R_Date("ISGS-A1328",690,20)
   {
    Outlier("General",0.05);
   };
   Interval("Interval Kirschner Early",LnN(ln(50),ln(2)));
   Date("Kirschner Early")
   {
    color="green";
   };
  };
 Boundary("End Kirschner Early");
};
Sequence()
{
 Boundary("Start Wylie 3");
 Phase ("Wylie 3")
  {
   R_Date("Beta-33183",780,60)
   {
    Outlier("Charcoal",1);
   };
   R_Date("Beta-33184",780,60)
   {
    Outlier("Charcoal",1);
   };
   //R_Date("Beta-33188",450,70){Outlier("Charcoal",1);};
   R_Date("Beta-40613",650,80)
   {
    Outlier("Charcoal",1);
   };
   R_Date("Beta-47106",720,80)
   {
    Outlier("Charcoal",1);
   };
   //R_Date("Beta-48223",400,70){Outlier("Charcoal",1);};
```

```
  R_Date("I-16767",600,80)
  {
   Outlier("Charcoal",1);
  };
  Interval("Interval Wylie 3",LnN(ln(50),ln(2)));
  Date("Wylie 3")
  {
   color="green";
  };
 };
 Boundary("End Wylie 3");
};
Sequence()
{
 Boundary("Start Morganza Reservoir");
 Phase("Morganza Reservoir")
 {
  R_Date("Beta-28096",820,50)
  {
   Outlier("Charcoal",1);
  };
  R_Date("Beta-28095",690,80)
  {
   Outlier("Charcoal",1);
  };
  R_Date("Beta-28094",600,60)
  {
   Outlier("Charcoal",1);
  };
  Interval("Interval Morganza Reservoir",LnN(ln(50),ln(2)));
  Date("Morganza Reservoir");
 };
 Boundary("End Morganza Reservoir");
};
Sequence()
{
 Boundary("Start Ashmore Farm");
 Phase("Ashmore Farm")
 {
  R_Date("ISGS-A-10696",525,45)
  {
   Outlier("General",0.05);
  };
  R_Date("ISGS-A-10697",570,80)
  {
   Outlier("General",0.05);
```

```
  };
  R_Date("ISGS-A10699",600,40)
  {
   Outlier("General",0.05);
  };
  R_Date("ISGS-A10700",575,40)
  {
   Outlier("General",0.05);
  };
  R_Date("ISGS-A10701",590,40)
  {
   Outlier("General",0.05);
  };
  //R_Date("ISGS-A10705",705,55){Outlier("General",0.05);};
  R_Date("ISGS-A10706",640,40)
  {
   Outlier("General",0.05);
  };
  R_Date("ISGS-A10707",630,50)
  {
   Outlier("General",0.05);
  };
  R_Date("ISGS-A10708",630,60)
  {
   Outlier("General",0.05);
  };
  //R_Date("Beta-48175",540,50){Outlier("General",0.05);};
  Interval("Interval Ashmore Farm",LnN(ln(50),ln(2)));
  Date("Ashmore Farm")
  {
   color="blue";
  };
 };
 Boundary("End Ashmore Farm");
};
Sequence()
{
 Boundary("Start Consol Early");
 Phase("Consol Early")
 {
  R_Date("ISGS-A1338,F460,maize",680,20)
  {
   Outlier("General",0.05);
  };
  R_Date("Beta-2040465,F468/B24,charcoal",570,60)
  {
```

```
   Outlier("Charcoal",1);
  };
  R_Date("ISGS-A4070,F1228,maize",610,20)
  {
   Outlier("General",0.05);
  };
  //R_Date("Beta-299410,F956,charcoal",540,30){Outlier("Charcoal",1);};
  Interval("Interval Consol Early",LnN(ln(50),ln(2)));
  Date("Consol Early")
  {
   color="green";
  };
 };
 Boundary("End Consol Early");
};
Sequence()
{
 Boundary("Start Gensler");
 Phase("Gensler")
 {
  R_Date("Dic-2599",630,65)
  {
   Outlier("Charcoal",1);
  };
  R_Date("Dic-2600",650,55)
  {
   Outlier("Charcoal",1);
  };
  R_Date("Dic-2601",690,50)
  {
   Outlier("Charcoal",1);
  };
  Interval("Interval Gensler",LnN(ln(50),ln(2)));
  Date("Gensler")
  {
   color="green";
  };
 };
 Boundary("End Gensler");
};
Sequence()
{
 Boundary("Start Portman");
 Phase("Portman")
 {
  R_Date("AA-38456",680,35)
```

```
   {
    Outlier("General",0.05);
   };
  R_Date("Beta-57298",560,50)
   {
    Outlier("Charcoal",1);
   };
  R_Date("UGa-1643",530,90)
   {
    Outlier("Charcoal",1);
   };
  Interval("Interval Portman",LnN(ln(50),ln(2)));
  Date("Portman")
   {
    color="red";
   };
  };
 Boundary("End Portman");
};
Sequence()
{
 Boundary("Start Goodwin-Portman");
 Phase("Goodwin-Portman")
  {
  R_Date("Beta-153248",510,60)
   {
    Outlier("Charcoal",1);
   };
  //R_Date("M-2203",740,100){Outlier("Charcoal",1);};
  R_Date("Beta-153000",680,60)
   {
    Outlier("Charcoal",1);
   };
  Interval("Interval Goodwin-Portman",LnN(ln(50),ln(2)));
  Date("Goodwin-Portman")
   {
    color="red";
   };
  };
 Boundary("End Goodwin-Portman");
};
Sequence()
{
 Boundary("Start Saddle Late");
 Phase("Saddle Late")
  {
```

```
  R_Combine("Feature 35")
  {
   Outlier("General",0.05);
   R_Date("AA-38457",675,33)
   {
    Outlier("SSimple",1);
   };
   R_Date("AA-38458",605,34)
   {
    Outlier("SSimple",1);
   };
  };
  R_Date("Beta-46465",550,50)
  {
   Outlier("Charcoal",1);
  };
  R_Date("Beta-46467",580,70)
  {
   Outlier("Charcoal",1);
  };
  R_Date("Beta-46468",590,50)
  {
   Outlier("Charcoal",1);
  };
  R_Date("Beta-46470",590,70)
  {
   Outlier("Charcoal",1);
  };
  Interval("Interval Saddle Late",LnN(ln(50),ln(2)));
  Date("Date Estimate Saddle Late")
  {
   color="red";
  };
 };
 Boundary("End Saddle Late");
};
Sequence()
{
 Boundary("Start Morganza Lang");
 Phase("Morganza Lang")
 {
  R_Date("Beta-19161",650,70)
  {
   Outlier("Charcoal",1);
  };
  //R_Date("Beta-19162",490,110){Outlier("Charcoal",1);};
```

```
  //R_Date("Beta-245150",410,40){Outlier("Charcoal",1);};
  R_Date("Beta-245152",660,40)
  {
   Outlier("Charcoal",1);
  };
  Interval("Interval Morganza Lang",LnN(ln(50),ln(2)));
  Date("Date Estimate Morganza Lang")
  {
   color="red";
  };
 };
 Boundary("End Morganza Lang");
};
Sequence()
{
 Boundary("Start Howarth-Nelson");
 Phase("Howarth-Nelson")
 {
  R_Date("PITT-38",530,35)
  {
   Outlier("Charcoal",1);
  };
  R_Date("PITT-37",530,35)
  {
   Outlier("Charcoal",1);
  };
  R_Date("PITT-36",580,40)
  {
   Outlier("Charcoal",1);
  };
  R_Date("PITT-39",505,35)
  {
   Outlier("Charcoal",1);
  };
  R_Date("PITT-274",625,50)
  {
   Outlier("Charcoal",1);
  };
  Interval("Interval Howarth-Nelson",LnN(ln(50),ln(2)));
  Date("Howarth-Nelson")
  {
   color="red";
  };
 };
 Boundary("End Howarth-Nelson");
};
```

```
Sequence()
{
 Boundary("Start Campbell Farm Late");
 Phase("Campbell Farm Late")
 {
  R_Date("AA-40132",462,38)
  {
   Outlier("General",0.05);
  };
  R_Date("DIC-2241",530,45)
  {
   Outlier("Charcoal",1);
  };
  R_Date("ISGS-A0773",515,35)
  {
   Outlier("Charcoal",1);
  };
  R_Date("ISGS-A0774",515,30)
  {
   Outlier("Charcoal",1);
  };
  //R_Date("M-2204",520,110){Outlier("Charcoal",1);};
  //R_Date("SI-1322",425,105){Outlier("Charcoal",1);};
  //R_Date("SI-1323",1010,95){Outlier("Charcoal",1);};
  R_Date("SI-3672",550,60)
  {
   Outlier("General",0.05);
  };
  Date("Campbell Farm Late")
  {
   color="blue";
  };
  Interval("Interval  Campbell Farm Late",LnN(ln(50),ln(2)));
 };
 Boundary("End  Campbell Farm Late");
};
Sequence()
{
 Boundary("Start Consol Late");
 Phase("Consol Late")
 {
  R_Date("Beta-164643 F49/B6",640,70)
  {
   Outlier("Charcoal",1);
  };
  R_Date("Beta-172012,F36/B4,charcoal",550,60)
```

```
  {
   Outlier("Charcoal",1);
  };
  R_Date("Beta-189627,F175/B8,charcoal",550,50)
  {
   Outlier("Charcoal",1);
  };
  R_Date("Beta-149289,F3/B1",540,50)
  {
   Outlier("Charcoal",1);
  };
  R_Date("Beta-172013,F130,charcoal",490,50)
  {
   Outlier("Charcoal",1);
  };
  R_Date("Beta-189128,F190,charcoal",470,70)
  {
   Outlier("Charcoal",1);
  };
  R_Combine("F266,House7Gh")
  {
   Outlier("General",0.05);
   R_Date("ISGS-A0767,bean",450,30)
   {
    Outlier("SSimple",0.05);
   };
   R_Date("ISGS-A0766,maize",405,30)
   {
    Outlier("SSimple",0.05);
   };
  };
  Interval("Interval Consol Late",LnN(ln(50),ln(2)));
  Date("Consol Late")
  {
   color="red";
  };
 };
 Boundary("End Consol Late");
};
Sequence()
{
 Boundary("Start Household");
 Phase("Household")
 {
  R_Date("I-16747",490,80)
  {
```

```
   Outlier("Charcoal",1);
  };
  R_Date("UGa-3453",325,80)
  {
   Outlier("Charcoal",1);
  };
  R_Date("Beta-249013",570,40)
  {
   Outlier("Charcoal",1);
  };
  Interval("Interval Household",LnN(ln(50),ln(2)));
  Date("Household")
  {
   color="orange";
  };
 };
 Boundary("End Household");
};
Sequence()
{
 Boundary("Start Kirschner Late");
 Phase("Kirschner Late")
 {
  R_Date("I-18577",490,80)
  {
   Outlier("General",0.05);
  };
  R_Date("ISGS-A1329",410,20)
  {
   Outlier("General",0.05);
  };
  R_Date("ISGS-A1330",400,20)
  {
   Outlier("General",0.05);
  };
  R_Date("ISGS-A1332",380,20)
  {
   Outlier("General",0.05);
  };
  R_Combine("House 13")
  {
   Outlier("General",0.05);
   R_Date("GX-21854",460,70)
   {
    Outlier("SSimple",0.05);
   };
```

```
   R_Date("I-18578",400,80)
   {
    Outlier("SSimple",0.05);
   };
  };
  //R_Date("ISGS-A0772",340,30){Outlier("General",0.05);};
  Interval("Interval Kirschner Late",LnN(ln(50),ln(2)));
  Date("Kirschner Late")
  {
   color="red";
  };
 };
 Boundary("End Kirschner Late");
};
Sequence()
{
 Boundary("Start Grays Landing 2");
 Phase("Grays Landing 2")
 {
  R_Date("UGa-5954",397,79)
  {
   Outlier("Charcoal",1);
  };
  R_Date("UGa-5955",559,50)
  {
   Outlier("Charcoal",1);
  };
  R_Date("UGa-5958",362,48)
  {
   Outlier("Charcoal",1);
  };
  R_Date("UGa-5959",299,99)
  {
   Outlier("Charcoal",1);
  };
  //R_Date("UGa-5960",490,103){Outlier("Charcoal",1);};
  R_Date("UGa-5962",460,88)
  {
   Outlier("Charcoal",1);
  };
  //R_Date("UGa-5964",254,58){Outlier("Charcoal",1);};
  R_Date("UGa-5965",663,91)
  {
   Outlier("Charcoal",1);
  };
  //R_Date("UGa-5968",600,126){Outlier("Charcoal",1);};
```

```
  R_Date("UGa-5969",467,70)
  {
   Outlier("Charcoal",1);
  };
  //R_Date("UGa-5970",467,100){Outlier("Charcoal",1);};
  //R_Date("UGa-5972",427,109){Outlier("Charcoal",1);};
  Interval("Interval Grays Landing 2",LnN(ln(50),ln(2)));
  Date("Grays Landing 2")
  {
   color="red";
  };
 };
 Boundary("End Grays Landing 2");
};
Sequence()
{
 Boundary("Start Mon City");
 Phase("Mon City")
 {
  R_Date("Beta-15280",330,50)
  {
   Outlier("Charcoal",1);
  };
  R_Date("Beta-15819",420,60)
  {
   Outlier("Charcoal",1);
  };
  R_Date("Beta-15821",530,70)
  {
   Outlier("Charcoal",1);
  };
  R_Date("Beta-15822",530,70)
  {
   Outlier("Charcoal",1);
  };
  Interval("Interval Mon City",LnN(ln(50),ln(2)));
  Date("Mon City")
  {
   color="red";
  };
 };
 Boundary("End Mon City");
};
Sequence()
{
 Boundary("Start Brokaw");
```

```
Phase("Brokaw")
{
 //R_Date("Dic-391",360,110){Outlier("Charcoal",1);};
 R_Date("Dic-392",740,55)
 {
  Outlier("Charcoal",1);
 };
 R_Date("N-3481",505,75)
 {
  Outlier("Charcoal",1);
 };
 R_Date("N-3482",525,75)
 {
  Outlier("Charcoal",1);
 };
 R_Date("N-3483",460,55)
 {
  Outlier("Charcoal",1);
 };
 //R_Date("TEM-167",530,120){Outlier("Charcoal",1);};
 //R_Date("TEM-168",720,100){Outlier("Charcoal",1);};
 R_Date("TEM-169",620,70)
 {
  Outlier("Charcoal",1);
 };
 R_Date("TEM-185",590,80)
 {
  Outlier("Charcoal",1);
 };
 R_Date("UGa-3429",405,65)
 {
  Outlier("Charcoal",1);
 };
 R_Date("UGa-3430",520,65)
 {
  Outlier("Charcoal",1);
 };
 R_Date("UGa-3943",390,70)
 {
  Outlier("Charcoal",1);
 };
 Interval("Interval Brokaw",LnN(ln(50),ln(2)));
 Date("Date Estimate Brokaw")
 {
  color="red";
 };
```

```
  };
 Boundary("End Brokaw");
};
Sequence()
{
 Boundary("Start Squirrel Hill");
 Phase("Squirrel Hill")
 {
  R_Date("ISGS-A2777",495,25)
  {
   Outlier("General",0.05);
  };
  R_Date("ISGS-A2776",410,20)
  {
   Outlier("General",0.05);
  };
  R_Date("Beta-339435",400,30)
  {
   Outlier("General",0.05);
  };
  Interval("Interval Squirrel Hill",LnN(ln(50),ln(2)));
  Date("Date Estimate Squirrel Hill")
  {
   color="red";
  };
 };
 Boundary("End Squirrel Hill");
};
Sequence()
{
 Boundary("Start Throckmorton");
 Phase("Throckmorton")
 {
  R_Date("Dic-2602",400,50)
  {
   Outlier("Charcoal",1);
  };
  R_Date("Dic-2604",300,60)
  {
   Outlier("Charcoal",1);
  };
  Interval("Interval Throckmorton",LnN(ln(50),ln(2)));
  Date("Throckmorton")
  {
   color="orange";
  };
```

```
  };
  Boundary("End Throckmorton");
 };
 Order("Order Sites");
};
```

Johnston site Model

```
Plot()
 {
 Outlier_Model("General",T(5),U(0,4),"t");
 Outlier_Model("Charcoal",Exp(1,-10,0),U(0,3),"t");
 Outlier_Model("SSimple",N(0,2),0,"s");
 Sequence()
 {
  Boundary("Start Johnston");
  Phase("Johnston")
  {
   //ISGS-A1759 was originally identified as a maize cupule recovered from a postmold.  It returned a δ13C  value of -25.9.
   //R_Date("ISGS-A1759, unknown",915,20){Outlier("Charcoal",1);};
   //R_Date("Beta-339596",840,30){Outlier("Charcoal",1);};
   R_Date("Beta-274127,maize",670,40)
   {
    Outlier("General",0.05);
   };
   R_Date("Beta-339431",650,30){Outlier("Charcoal",1);};
   R_Date("Beta-206729",630,40)
   {
    Outlier("Charcoal",1);
   };
   R_Date("ISGS-A1736,maize",610,20)
   {
    Outlier("General",0.05);
   };
   R_Date("Beta-274125,maize",590,40)
   {
    Outlier("General",0.05);
   };
   R_Date("Beta-228173",590,40)
   {
    Outlier("Charcoal",1);
   };
   R_Date("Beta-339434",560,30)
   {
    Outlier("Charcoal",1);
   };
```

```
//R_Date("ISGS-A1508,maize",565,15)
//{
//Outlier("General",0.05);
//};
R_Date("Beta-439412",540,30)
{
 Outlier("Charcoal",1);
};
R_Date("ISGS-A3788",535,20)
{
 Outlier("Charcoal",1);
};
R_Date("Beta-274120,maize",530,40)
{
 Outlier("General",0.5);
};
R_Date("Beta-274123,gourd",500,40)
{
 Outlier("General",0.5);
};
R_Date("Beta-274122",490,40)
{
 Outlier("Charcoal",1);
};
R_Date("Beta-226700",460,50)
{
 Outlier("Charcoal",1);
};
R_Date("Beta-228176",460,40)
{
 Outlier("Charcoal",1);
};
R_Date("Beta-265175",460,40)
{
 Outlier("Charcoal",1);
};
R_Date("ISGS-A2115",455,20)
{
 Outlier("Charcoal",1);
};
R_Combine("Feature 28")
{
 Outlier("General",0.05);
 R_Date("ISGS-A1506,seed",450,15)
  {
  Outlier("SSimple",0.05);
```

```
 };
 R_Date("Beta-254453,maize",510,40)
 {
  Outlier("SSimple",0.05);
 };
};
R_Date("Beta-274124,gourd",430,20)
{
 Outlier("General",0.05);
};
R_Date("Beta-439411",420,30)
{
 Outlier("Charcoal",1);
};
R_Date("ISGS-A3787,maize",420,20)
{
 Outlier("General",0.05);
};
R_Date("ISGS-A2116,maize",420,20)
{
 Outlier("General",0.05);
};
R_Date("ISGS-A1970,bean",420,15)
{
 Outlier("General",0.05);
};
R_Date("ISGS-A5945,maize",415,25)
{
 Outlier("General",0.05);
};
R_Combine("Feature 109")
{
 Outlier("General",0.05);
 R_Date("ISGS-A1507,seed",410,15)
 {
  Outlier("SSimple",0.05);
 };
 R_Date("ISGS-A1509,maize",420,15)
 {
  Outlier("SSimple",0.05);
 };
 R_Date("ISGS-A1510,nutshell",420,15)
 {
  Outlier("SSimple",0.05);
 };
};
```

```
  R_Date("ISGS-A2878,maize",405,25)
  {
   Outlier("General",0.05);
  };
  R_Date("ISGS-A2877,maize",395,25)
  {
   Outlier("General",0.05);
  };
  R_Date("Beta-274121,maize",390,40)
  {
   Outlier("General",0.05);
  };
  R_Combine("Feature 198")
  {
   Outlier("General",0.05);
   R_Date("ISGS-A1969,bean",380,15)
   {
    Outlier("SSimple",0.05);
   };
   R_Date("ISGS-A1737,maize",400,20)
   {
    Outlier("SSimple",0.05);
   };
  };
  //Stockade posts not included in the model because most have obvious large inbuilt ages.
  //R_Date("Beta-253131",1310,60){Outlier("Charcoal",1);};
  //R_Date("Beta-228174",1060,50){Outlier("Charcoal",1);};
  //R_Date("Beta-228175",960,50){Outlier("Charcoal",1);};
  //R_Date("Beta-255089",1310,40){Outlier("Charcoal",1);};
  //R_Date("Beta-255090",990,40){Outlier("Charcoal",1);};
  //R_Date("Beta-339597",970,30){Outlier("Charcoal",1);};
  //R_Date("Beta-255825",850,50){Outlier("Charcoal",1);};
  //R_Date("Beta-228174",720,40){Outlier("Charcoal",1);};
  Interval("Interval Johnston");
  Date("Date Estimate Johnston");
 };
 Boundary("End Johnston");
};
};
```

OxCal Code for Allegheny Mountains Section Sites

Fort Hill Site

```
Plot()
 {
  Outlier_Model("General",T(5),U(0,4),"t");
  Outlier_Model("Charcoal",Exp(1,-10,0),U(0,3),"t");
  Outlier_Model("SSimple",N(0,2),0,"s");
  Sequence()
  {
   Boundary("Start Fort Hill I");
   Phase("Fort Hill I")
   {
    //R_Date("AA-58578",990,69)
    //{
     //Outlier("General",1);
    //};
    R_Date("Beta-217462",830,40)
    {
     Outlier("Charcoal",1);
    };
    R_Combine("Feature 49")
    {
     Outlier("General",0.05);
     R_Date("AA-53667a",802,34)
     {
      Outlier("SSimple",0.05);
     };
     R_Date("AA-53667b",847,35)
     {
      Outlier("SSimple",0.05);
     };
    };
    R_Date("AA-53663",763,43)
    {
     Outlier("General",0.05);
    };
    R_Date("Beta-217461",760,40)
    {
     Outlier("Charcoal",1);
    };
    R_Date("AA-53669",754,34)
    {
     Outlier("General",0.05);
    };
```

```
 R_Date("AA-53672",753,41)
 {
  Outlier("Charcoal",1);
 };
 R_Date("Beta-217466",730,50)
 {
  Outlier("Charcoal",1);
 };
 Interval("Interval Fort Hill I",N(50,10));
 Date("Date Estimate Fort Hill I");
};
Boundary("End Fort Hill I");
Interval("Interval between Fort Hill I and Fort Hill II");
Boundary("Start Fort Hill II");
Phase("Fort Hill II")
{
 R_Date("Beta-217459",720,40)
 {
  Outlier("Charcoal",1);
 };
 R_Date("Beta-217464",710,40)
 {
  Outlier("Charcoal",1);
 };
 R_Date("AA-53659",695,38)
 {
  Outlier("General",0.05);
 };
 R_Date("AA-53673",658,33)
 {
  Outlier("General",0.05);
 };
 R_Date("AA-53664",693,39)
 {
  Outlier("General",0.05);
 };
 R_Date("Beta-217458",670,40)
 {
  Outlier("Charcoal",1);
 };
 R_Date("Beta-217457",660,40)
 {
  Outlier("Charcoal",1);
 };
 R_Date("Beta-217465",630,50)
 {
```

```
    Outlier("Charcoal",1);
   };
   R_Date("Beta-217463",640,40)
   {
    Outlier("Charcoal",1);
   };
   //R_Date("AA-53666",582,57){Outlier("General",0.05);};
   //R_Date("Beta-217460",480,40){Outlier("Charcoal",1);};
   //R_Date("AA-53670",366,46){Outlier("Charcoal",1);};
   Interval("Interval Fort Hill II",N(50,10));
   Date("Date Estimate Fort Hill II");
  };
  Boundary("End Fort Hill II");
 };
};
```

Gnagey Site

```
Plot()
 {
  Outlier_Model("General",T(5),U(0,4),"t");
  Outlier_Model("Charcoal",Exp(1,-10,0),U(0,3),"t");
  Sequence("Gnagey 3")
  {
   Boundary("Start Inner");
   Phase("Inner")
   {
    R_Date("AA-29117",610,55)
    {
     Outlier("General",0.05);
    };
    R_Date("AA-53306",713,34)
    {
     Outlier("General",0.05);
    };
    R_Date("AA-53310",692,46)
    {
     Outlier("General",0.05);
    };
    R_Date("Beta-242610",880,40)
    {
     Outlier("Charcoal",1);
    };
    R_Date("Beta-242611",790,40)
    {
     Outlier("Charcoal",1);
    };
```

```
   R_Date("Beta-242613",810,40)
   {
    Outlier("Charcoal",1);
   };
   R_Date("UGa-1599",945,80)
   {
    Outlier("Charcoal",1);
   };
   Interval("Interval Inner",N(50,10));
   Date("Date Estimate Inner");
  };
  Boundary("End Inner");
  Interval("Interval Between Inner and Outer");
  Boundary("Start Outer");
  Phase("Outer")
  {
   R_Date("AA-29118",635,45)
   {
    Outlier("General",0.05);
   };
   R_Date("AA-53311",590,50)
   {
    Outlier("General",0.05);
   };
   Interval("Interval Outer",N(50,10));
   Date("Date Estimate Outer");
  };
  Boundary("End Outer");
 };
};
```

Peck No. 1

```
Plot()
 {
  Outlier_Model("General",T(5),U(0,4),"t");
  Outlier_Model("Charcoal",Exp(1,-10,0),U(0,3),"t");
  Outlier_Model("SSimple",N(0,2),0,"s");
  Sequence("Peck No. 1")
  {
   Boundary("Start Initial Core");
   Phase("Initial Core")
   {
    R_Date("AA-52970", 639, 44)
    {
     Outlier("Charcoal",1);
```

```
  };
  R_Date("AA-52971",578,42)
  {
   Outlier("Charcoal",1);
  };
  R_Date("AA-53678",737,46)
  {
   Outlier("Charcoal",1);
  };
  Interval("Interval Initial Core",N(50,10));
  Date("Date Estimate Initial Core");
 };
 Boundary("End Initial Core");
 Interval("Interval between Core 1 and Expansion 1");
 Boundary("StartExpansion 1");
 Phase("Expansion 1")
 {
  R_Date("AA-52972",762,36)
  {
   Outlier("Charcoal",1);
  };
  R_Date("AA-53667",614,35)
  {
   Outlier("Charcoal",1);
  };
  Interval("Interval Expansion 1",N(50,10));
  Date("Date Estimate Expansion 1");
 };
 Boundary("End Expansion 1");
 Interval("Interval between Expansion 1 and Expansion 2");
 Boundary("Start Expansion 2");
 Phase("Expansion 2")
 {
  R_Date("AA-52974",658,35)
  {
   Outlier("Charcoal",1);
  };
  R_Combine("Feature 103")
  {
   Outlier("General",0.05);
   R_Date("AA-53667",540,36)
   {
    Outlier("SSimple",0.05);
   };
   R_Date("AA-52973",536,36)
   {
```

```
     Outlier("SSimple",0.05);
    };
   };
   Interval("Interval Expansion 2",N(50,10));
   Date("Date Estimate Expansion 2");
  };
  Boundary("End Expansion 2");
 };
};
```

Peck No. 2

```
Plot()
 {
  Outlier_Model("General",T(5),U(0,4),"t");
  Outlier_Model("Charcoal",Exp(1,-10,0),U(0,3),"t");
  Sequence("Peck No. 2")
  {
   Boundary("Start First");
   Phase("First")
   {
    R_Date("AA-52976", 889, 38)
    {
     Outlier("Charcoal",1);
    };
    R_Date("AA-52977",864,40)
    {
     Outlier("Charcoal",1);
    };
    R_Date("AA-52678",918,39)
    {
     Outlier("Charcoal",1);
    };
    R_Date("AA-52975",815,36)
    {
     Outlier("Charcoal",1);
    };
    R_Date("AA-52966",913,46)
    {
     Outlier("Charcoal",1);
    };
    Interval("Interval First",N(50,10));
    Date("Date Estimate First");
   };
   Boundary("End First");
   Interval("Interval between First and Second");
   Boundary("Start Second");
```

```
  Phase("Second")
  {
   R_Date("AA-53309",364,44)
   {
    Outlier("General",0.05);
   };
   R_Date("AA-53309",321,41)
   {
    Outlier("Charcoal",1);
   };
   R_Date("AA-52968",561,42)
   {
    Outlier("Charcoal",1);
   };
   R_Date("AA-52969",418,42)
   {
    Outlier("Charcoal",1);
   };
   Interval("Interval Second",N(50,10));
   Date("Date Estimate Second");
  };
  Boundary("End Second");
 };
};
```

Petenbrink

```
Plot()
 {
 Outlier_Model("Charcoal",Exp(1,-10,0),U(0,3),"t");
 Sequence()
 {
  Boundary("Start Petenbrink 1");
  Phase("Petenbrink 1")
  {
   //R_Date("Beta-104103",1080,70){Outlier("Charcoal",1);};
   R_Date("Beta-776777",1010,60)
   {
    Outlier("Charcoal",1);
   };
   R_Date("Beta-104100",940,70)
   {
    Outlier("Charcoal",1);
   };
   R_Date("Beta-104102",940,50)
   {
```

```
  Outlier("Charcoal",1);
 };
 R_Date("Beta-104104",930,50)
 {
  Outlier("Charcoal",1);
 };
 R_Date("Beta-104107",920,60)
 {
  Outlier("Charcoal",1);
 };
 R_Date("Beta-104108",930,70)
 {
  Outlier("Charcoal",1);
 };
 R_Date("Beta-104112",890,70)
 {
  Outlier("Charcoal",1);
 };
 Interval("Interval Petenbrink 1",N(50,10));
 Date("Date Petenbrink 1");
};
Boundary("End Petenbrink 1");
Interval("Interval between Petenbrink 1 and Petenbrink 2");
Boundary("Start Petenbrink 2");
Phase("Petenbrink 2")
{
 R_Date("Beta-104109",830,70)
 {
  Outlier("Charcoal",1);
 };
 R_Date("Beta-104110",710,60)
 {
  Outlier("Charcoal",1);
 };
 R_Date("Beta-104111",790,60)
 {
  Outlier("Charcoal",1);
 };
 R_Date("Beta-104115",820,50)
 {
  Outlier("Charcoal",1);
 };
 R_Date("Beta-104106",820,50)
 {
  Outlier("Charcoal",1);
 };
```

```
   Interval("Interval Petenbrink 2",N(50,10));
   Date("Date Petenbrink 2");
  };
  Boundary("End Petenbrink 2");
 };
};
```

Railroad Site

```
Plot()
 {
 Outlier_Model("Charcoal",Exp(1,-10,0),U(0,3),"t");
 Sequence("Railroad")
 {
  Boundary("Start Railroad I");
  Phase("Railroad I")
  {
   R_Date("Beta-104118",820,60)
   {
    Outlier("Charcoal",1);
   };
   R_Date("Beta-104119",860,80)
   {
    Outlier("Charcoal",1);
   };
   //R_Date("Beta-104120",960,60)
   //{
   //Outlier("Charcoal",1);
   //};
   R_Date("Beta-104121",780,60)
   {
    Outlier("Charcoal",1);
   };
   //R_Date("Beta-104122",900,60)
   //{
   //Outlier("Charcoal",1);
   //};
   R_Date("Beta-104123",840,60)
   {
    Outlier("Charcoal",1);
   };
   R_Date("Beta-104125",730,50)
   {
    Outlier("Charcoal",1);
   };
   R_Date("Beta-104128",760,60)
   {
```

```
  Outlier("Charcoal",1);
 };
 R_Date("Beta-104129",770,80)
 {
  Outlier("Charcoal",1);
 };
 R_Date("Beta-104130",830,80)
 {
  Outlier("Charcoal",1);
 };
 //R_Date("Beta-104131",960,70)
 //{
 //Outlier("Charcoal",1);
 //};
 //R_Date("Beta-104134",970,80)
 //{
 //Outlier("Charcoal",1);
 //};
 R_Date("Beta-104135",850,60)
 {
  Outlier("Charcoal",1);
 };
 R_Date("Beta-77680",880,80)
 {
  Outlier("Charcoal",1);
 };
 R_Date("Beta-92578",730,50)
 {
  Outlier("Charcoal",1);
 };
 Interval("Interval Railroad I",N(50,10));
 Date("Date Estimate Railroad I");
};
Boundary("End Railroad I");
Interval("Interval between Railroad I and Railroad II");
Boundary("Start Railroad II");
Phase("Railroad II")
{
 R_Date("Beta-104126",510,50)
 {
  Outlier("Charcoal",1);
 };
 R_Date("Beta-104127",650,60)
 {
  Outlier("Charcoal",1);
 };
```

```
   R_Date("Beta-104132",550,60)
   {
    Outlier("Charcoal",1);
   };
   R_Date("Beta-96146",550,90)
   {
    Outlier("Charcoal",1);
   };
   Interval("Interval Railroad II",N(50,10));
   Date("Date Estimate Railroad II");
  };
  Boundary("End Railroad II");
 };
};
```

Allegheny Mountains section Model 1

```
Plot()
 {
  Outlier_Model("General",T(5),U(0,4),"t");
  Outlier_Model("Charcoal",Exp(1,-10,0),U(0,3),"t");
  Outlier_Model("SSimple",N(0,2),0,"s");
  Phase ()
  {
   Sequence()
   {
    Boundary("Start Petenbrink 1");
    Phase("Second Petenbrink 1")
    {
    //R_Date("Beta-104103",1080,70){Outlier("Charcoal",1);};
     R_Date("Beta-776777",1010,60)
     {
      Outlier("Charcoal",1);
     };
     R_Date("Beta-104100",940,70)
     {
      Outlier("Charcoal",1);
     };
     R_Date("Beta-104102",940,50)
     {
      Outlier("Charcoal",1);
     };
     R_Date("Beta-104104",930,50)
     {
      Outlier("Charcoal",1);
     };
     R_Date("Beta-104107",920,60)
```

```
  {
   Outlier("Charcoal",1);
  };
  R_Date("Beta-104108",930,70)
  {
   Outlier("Charcoal",1);
  };
  R_Date("Beta-104112",890,70)
  {
   Outlier("Charcoal",1);
  };
  Interval("Interval Petenbrink 1",N(50,10));
  Date("Date Petenbrink 1");
 };
 Boundary("End Petenbrink 1");
};
Sequence()
{
 Boundary("Start Peck No 2 Early");
 Phase ("Peck No 2 Early")
 {
  R_Date("AA-52976",889,38)
  {
   Outlier("General",0.05);
  };
  R_Date("AA-52977",864,40)
  {
   Outlier("General",0.05);
  };
  R_Date("AA-52978",918,39)
  {
   Outlier("General",0.05);
  };
  Interval("Interval Peck No 2 Early",N(50,10));
  Date("Date Estimate Peck No 2 Early");
 };
 Boundary("End Peck No 2 Early");
};
Sequence()
{
 Boundary("Start Railroad Early");
 Phase("Railroad Early")
 {
  R_Date("Beta-104118",820,80)
  {
   Outlier("Charcoal",1);
```

```
};
R_Date("Beta-104119",860,80)
{
 Outlier("Charcoal",1);
};
//R_Date("Beta-104120",960,60){Outlier("Charcoal",1);};
R_Date("Beta-104121",780,50)
{
 Outlier("Charcoal",1);
};
//R_Date("Beta-104122",900,60){Outlier("Charcoal",1);};
R_Date("Beta-104123",840,60)
{
 Outlier("Charcoal",1);
};
R_Date("Beta-104125",730,50)
{
 Outlier("Charcoal",1);
};
R_Date("Beta-104128",760,60)
{
 Outlier("Charcoal",1);
};
R_Date("Beta-104129",770,80)
{
 Outlier("Charcoal",1);
};
R_Date("Beta-104130",830,80)
{
 Outlier("Charcoal",1);
};
//R_Date("Beta-104131",960,70){Outlier("Charcoal",1);};
//R_Date("Beta-104134",970,80){Outlier("Charcoal",1);};
R_Date("Beta-104135",850,60)
{
 Outlier("Charcoal",1);
};
R_Date("Beta-77680",880,80)
{
 Outlier("Charcoal",1);
};
R_Date("Beta-92578",730,50)
{
 Outlier("Charcoal",1);
};
Interval("Interval Railroad Early",N(50,10));
```

```
  Date("Date Estimate Railroad Early");
 };
 Boundary("End Railroad Early");
};
Sequence()
{
 Boundary("Start Sang Run");
 Phase("Sang Run")
 {
  R_Date("Beta-24720",770,60)
  {
   Outlier("Charcoal",1);
  };
  R_Date("Beta-24721",830,60)
  {
   Outlier("Charcoal",1);
  };
  Interval("Interval Sang Run",N(50,10));
  Date("Date Estimate Sang Run");
 };
 Boundary("End Sang Run");
};
Sequence()
{
 Boundary("Start Petenbrink 2");
 Phase("Petenbrink 2")
 {
  R_Date("Beta-104109",830,70)
  {
   Outlier("Charcoal",1);
  };
  R_Date("Beta-104110",710,60)
  {
   Outlier("Charcoal",1);
  };
  R_Date("Beta-104111",790,60)
  {
   Outlier("Charcoal",1);
  };
  R_Date("Beta-104115",820,50)
  {
   Outlier("Charcoal",1);
  };
  R_Date("Beta-104106",820,50)
  {
   Outlier("Charcoal",1);
```

```
  };
  Interval("Interval Petenbrink 2",N(50,10));
  Date("Date Petenbrink 2");
 };
 Boundary("End Petenbrink 2");
};
Sequence()
{
 Boundary("Start Fort Hill");
 Phase("Fort Hill")
 {
  R_Date("AA-53659",695,38)
  {
   Outlier("General",0.05);
  };
  R_Date("AA-53663",763,43)
  {
   Outlier("General",0.05);
  };
  R_Date("AA-53664",693,39)
  {
   Outlier("General",0.05);
  };
  R_Date("AA-53666",582,57)
  {
   Outlier("General",0.05);
  };
  //R_Date("AA-53667a",847,35){Outlier("SSimple",0.05);};
  R_Date("AA-53667b",802,34)
  {
   Outlier("SSimple",0.05);
  };
  R_Date("AA-53669",754,34)
  {
   Outlier("General",0.05);
  };
  R_Date("AA-53672",753,41)
  {
   Outlier("General",0.05);
  };
  R_Date("AA-53673",658,33)
  {
   Outlier("General",0.05);
  };
  //R_Date("AA-58578",990,69)
  //{
```

```
  //Outlier("General",0.05);
  //};
  R_Date("Beta-217457",660,40)
  {
   Outlier("Charcoal",1);
  };
  R_Date("Beta-217466",730,50)
  {
   Outlier("Charcoal",1);
  };
  R_Date("Beta-217458",670,40)
  {
   Outlier("Charcoal",1);
  };
  //R_Date("Beta-217460",480,40){Outlier("Charcoal",1);};
  R_Date("Beta-217461",760,40)
  {
   Outlier("Charcoal",1);
  };
  R_Date("Beta-217462",830,40)
  {
   Outlier("Charcoal",1);
  };
  R_Date("Beta-217463",640,40)
  {
   Outlier("Charcoal",1);
  };
  R_Date("Beta-217464",710,40)
  {
   Outlier("Charcoal",1);
  };
  R_Date("Beta-217465",630,40)
  {
   Outlier("Charcoal",1);
  };
  Interval("Interval Fort Hill",N(50,10));
  Date("Date Fort Hill");
 };
 Boundary("End Fort Hill");
};
Sequence()
{
 Boundary("Start Gnagey No 3");
 Phase("Gnagey 3")
 {
  R_Date("AA-29117",610,55)
```

```
   {
    Outlier("General",0.05);
   };
   R_Date("AA-29118",635,45)
   {
    Outlier("General",0.05);
   };
   R_Date("AA-53306",713,34)
   {
    Outlier("General",0.05);
   };
   R_Date("AA-53310",692,46)
   {
    Outlier("General",0.05);
   };
   R_Date("AA-53311",590,50)
   {
    Outlier("General",0.05);
   };
   //R_Date("Beta-242610",880,40){Outlier("Charcoal",1);};
   R_Date("Beta-242611",790,40)
   {
    Outlier("Charcoal",1);
   };
   R_Date("Beta-242613",810,40)
   {
    Outlier("Charcoal",1);
   };
   Interval("Interval Gnagey 3",N(50,10));
   Date("Date Estimate Gnagey 3");
  };
  Boundary("End Gnagey 3");
 };
 Sequence()
 {
  Boundary("Start Peck No 1");
  Phase("Peck No 1")
  {
   //R_Combine("Feature103"){Outlier("General",0.05);
   //R_Date("AA-52973",540,36){Outlier("SSimple",0.05);};
   //R_Date("AA-52973b", 536, 36){Outlier("SSimple",0.05);};
   //};
   R_Date("AA-52972",762,36)
   {
    Outlier("General",0.05);
   };
```

```
  R_Date("AA-52974",658,35)
  {
   Outlier("General",0.05);
  };
  R_Date("AA-53677",614,35)
  {
   Outlier("General",0.05);
  };
  R_Date("AA-53678",737,46)
  {
   Outlier("General",0.05);
  };
  Interval("Interval Peck No 1",N(50,10));
  Date("Date Estimate Peck No 1");
 };
 Boundary("End Peck No 1");
};
Sequence()
{
 Boundary("Start Jonas Field");
 Phase("Jonas Field")
 {
  R_Date("Beta-101492",590,60)
  {
   Outlier("Charcoal",1);
  };
  R_Date("Beta-85304",680,50)
  {
   Outlier("Charcoal",1);
  };
  R_Date("Beta-85305",780,60)
  {
   Outlier("Charcoal",1);
  };
  Interval("Interval Jonas Field",N(50,10));
  Date("Date Estimate Jonas Field");
 };
 Boundary("End Jonas Field");
};
Sequence()
{
 Boundary("Start Reckner");
 Phase("Reckner")
 {
  R_Combine("Feature 77")
  {
```

```
   Outlier("General",0.05);
   R_Date("AA-53662",599,43)
   {
    Outlier("SSimple",0.05);
   };
   R_Date("AA-53662b",529,40)
   {
    Outlier("SSimple",0.05);
   };
  };
  R_Date("AA-53676",583,34)
  {
   Outlier("General",0.05);
  };
  Interval("Interval Reckner",N(50,10));
  Date("Date Estimate Reckner");
 };
 Boundary("End Reckner");
};
Sequence()
{
 Boundary("Start Railroad Late");
 Phase("Railroad Late")
 {
  R_Date("Beta-104126",510,50)
  {
   Outlier("Charcoal",1);
  };
  R_Date("Beta-104127",650,60)
  {
   Outlier("Charcoal",1);
  };
  R_Date("Beta-104132",550,60)
  {
   Outlier("Charcoal",1);
  };
  R_Date("Beta-96146",550,90)
  {
   Outlier("Charcoal",1);
  };
  Interval("Interval Railroad Late",N(50,10));
  Date("Date Estimate Railroad Late");
 };
 Boundary("End Railroad Late");
};
Sequence()
```

```
   {
    Boundary("Start Peck No 2 Late");
    Phase("Peck No 2 Late")
    {
     R_Date("AA-53309",364,44)
     {
      Outlier("Charcoal",1);
     };
     R_Date("AA-52967",321,41)
     {
      Outlier("Charcoal",1);
     };
     //R_Date("AA-52968",561,42){Outlier("Charcoal",1);};
     R_Date("AA-52969",418,42)
     {
      Outlier("Charcoal",1);
     };
     Interval("Interval Peck No 2 Late",N(50,10));
     Date("Date Peck No 2 Late");
    };
    Boundary("End Peck No 2 Late");
   };
   Order("Order Sites");
  };
 };
```

Allegheny Mountains section Model 2

```
Plot()
 {
  Outlier_Model("General",T(5),U(0,4),"t");
  Outlier_Model("Charcoal",Exp(1,-10,0),U(0,3),"t");
  Outlier_Model("SSimple",N(0,2),0,"s");
  Phase ()
  {
   Sequence()
   {
    Boundary("Start Petenbrink early");
    Phase("Second Petenbrink early")
    {
     //R_Date("Beta-104103",1080,70){Outlier("Charcoal",1);};
     R_Date("Beta-776777",1010,60)
     {
      Outlier("Charcoal",1);
     };
     R_Date("Beta-104100",940,70)
     {
```

```
   Outlier("Charcoal",1);
  };
  R_Date("Beta-104102",940,50)
  {
   Outlier("Charcoal",1);
  };
  R_Date("Beta-104104",930,50)
  {
   Outlier("Charcoal",1);
  };
  R_Date("Beta-104107",920,60)
  {
   Outlier("Charcoal",1);
  };
  R_Date("Beta-104108",930,70)
  {
   Outlier("Charcoal",1);
  };
  R_Date("Beta-104112",890,70)
  {
   Outlier("Charcoal",1);
  };
  Interval("Interval Petenbrink early",LnN(ln(50),ln(2)));
  Date("Date Petenbrink early");
 };
 Boundary("End Petenbrink early");
};
Sequence()
{
 Boundary("Start Peck No 2 early");
 Phase ("Peck No 2 early")
 {
  R_Date("AA-52976",889,38)
  {
   Outlier("General",0.05);
  };
  R_Date("AA-52977",864,40)
  {
   Outlier("General",0.05);
  };
  R_Date("AA-52978",918,39)
  {
   Outlier("General",0.05);
  };
  Interval("Interval Peck No 2 early",LnN(ln(50),ln(2)));
  Date("Date Estimate Peck No 2 early");
```

```
 };
 Boundary("End Peck No 2 early");
};
Sequence()
{
 Boundary("Start Railroad Early");
 Phase("Railroad Early")
 {
  R_Date("Beta-104118",820,80)
  {
   Outlier("Charcoal",1);
  };
  R_Date("Beta-104119",860,80)
  {
   Outlier("Charcoal",1);
  };
  //R_Date("Beta-104120",960,60){Outlier("Charcoal",1);};
  R_Date("Beta-104121",780,50)
  {
   Outlier("Charcoal",1);
  };
  //R_Date("Beta-104122",900,60){Outlier("Charcoal",1);};
  R_Date("Beta-104123",840,60)
  {
   Outlier("Charcoal",1);
  };
  R_Date("Beta-104125",730,50)
  {
   Outlier("Charcoal",1);
  };
  R_Date("Beta-104128",760,60)
  {
   Outlier("Charcoal",1);
  };
  R_Date("Beta-104129",770,80)
  {
   Outlier("Charcoal",1);
  };
  R_Date("Beta-104130",830,80)
  {
   Outlier("Charcoal",1);
  };
  //R_Date("Beta-104131",960,70){Outlier("Charcoal",1);};
  //R_Date("Beta-104134",970,80){Outlier("Charcoal",1);};
  R_Date("Beta-104135",850,60)
  {
```

```
   Outlier("Charcoal",1);
  };
  R_Date("Beta-77680",880,80)
  {
   Outlier("Charcoal",1);
  };
  R_Date("Beta-92578",730,50)
  {
   Outlier("Charcoal",1);
  };
  Interval("Interval Railroad early",LnN(ln(50),ln(2)));
  Date("Date Estimate Railroad early");
 };
 Boundary("End Railroad early");
};
Sequence()
{
 Boundary("Start Sang Run");
 Phase("Sang Run")
 {
  R_Date("Beta-24720",770,60)
  {
   Outlier("Charcoal",1);
  };
  R_Date("Beta-24721",830,60)
  {
   Outlier("Charcoal",1);
  };
  Interval("Interval Sang Run",LnN(ln(50),ln(2)));
  Date("Date Estimate Sang Run");
 };
 Boundary("End Sang Run");
};
Sequence()
{
 Boundary("Start Petenbrink late");
 Phase("Petenbrink late")
 {
  R_Date("Beta-104109",830,70)
  {
   Outlier("Charcoal",1);
  };
  R_Date("Beta-104110",710,60)
  {
   Outlier("Charcoal",1);
  };
```

```
  R_Date("Beta-104111",790,60)
  {
   Outlier("Charcoal",1);
  };
  R_Date("Beta-104115",820,50)
  {
   Outlier("Charcoal",1);
  };
  R_Date("Beta-104106",820,50)
  {
   Outlier("Charcoal",1);
  };
  Interval("Interval Petenbrink late",LnN(ln(50),ln(2)));
  Date("Date Petenbrink late");
 };
 Boundary("End Petenbrink late");
};
Sequence()
{
 Boundary("Start Fort Hill");
 Phase("Fort Hill")
 {
  R_Date("AA-53659",695,38)
  {
   Outlier("General",0.05);
  };
  R_Date("AA-53663",763,43)
  {
   Outlier("General",0.05);
  };
  R_Date("AA-53664",693,39)
  {
   Outlier("General",0.05);
  };
  R_Date("AA-53666",582,57)
  {
   Outlier("General",0.05);
  };
  R_Combine("Feature 49")
  {
   Outlier("General",0.05);
   R_Date("AA-53667a",847,35)
   {
    Outlier("SSimple",0.05);
   };
   R_Date("AA-53667b",802,34)
```

```
  {
   Outlier("SSimple",0.05);
  };
 };
 R_Date("AA-53669",754,34)
 {
  Outlier("General",0.05);
 };
 R_Date("AA-53672",753,41)
 {
  Outlier("General",0.05);
 };
 R_Date("AA-53673",658,33)
 {
  Outlier("General",0.05);
 };
 //R_Date("AA-58578",990,69){Outlier("General",0.05);};
 R_Date("Beta-217457",660,40)
 {
  Outlier("Charcoal",1);
 };
 R_Date("Beta-217466",730,50)
 {
  Outlier("Charcoal",1);
 };
 R_Date("Beta-217458",670,40)
 {
  Outlier("Charcoal",1);
 };
 //R_Date("Beta-217460",480,40){Outlier("Charcoal",1);};
 R_Date("Beta-217461",760,40)
 {
  Outlier("Charcoal",1);
 };
 R_Date("Beta-217462",830,40)
 {
  Outlier("Charcoal",1);
 };
 R_Date("Beta-217463",640,40)
 {
  Outlier("Charcoal",1);
 };
 R_Date("Beta-217464",710,40)
 {
  Outlier("Charcoal",1);
 };
```

```
  R_Date("Beta-217465",630,40)
  {
   Outlier("Charcoal",1);
  };
  Interval("Interval Fort Hill",LnN(ln(50),ln(2)));
  Date("Date Fort Hill");
 };
 Boundary("End Fort Hill");
};
Sequence()
{
 Boundary("Start Gnagey No 3");
 Phase("Gnagey 3")
 {
  R_Date("AA-29117",610,55)
  {
   Outlier("General",0.05);
  };
  R_Date("AA-29118",635,45)
  {
   Outlier("General",0.05);
  };
  R_Date("AA-53306",713,34)
  {
   Outlier("General",0.05);
  };
  R_Date("AA-53310",692,46)
  {
   Outlier("General",0.05);
  };
  R_Date("AA-53311",590,50)
  {
   Outlier("General",0.05);
  };
  //R_Date("Beta-242610",880,40){Outlier("Charcoal",1);};
  R_Date("Beta-242611",790,40)
  {
   Outlier("Charcoal",1);
  };
  R_Date("Beta-242613",810,40)
  {
   Outlier("Charcoal",1);
  };
  Interval("Interval Gnagey 3",LnN(ln(50),ln(2)));
  Date("Date Estimate Gnagey 3");
 };
```

```
 Boundary("End Gnagey 3");
};
Sequence()
{
 Boundary("Start Peck No 1");
 Phase("Peck No 1")
 {
  //R_Combine("Feature103"){Outlier("General",0.05);
  //R_Date("AA-52973",540,36){Outlier("SSimple",0.05);};
  //R_Date("AA-52973b", 536, 36){Outlier("SSimple",0.05);};
  //};
  R_Date("AA-52972",762,36)
  {
   Outlier("General",0.05);
  };
  R_Date("AA-52974",658,35)
  {
   Outlier("General",0.05);
  };
  R_Date("AA-53677",614,35)
  {
   Outlier("General",0.05);
  };
  R_Date("AA-53678",737,46)
  {
   Outlier("General",0.05);
  };
  Interval("Interval Peck No 1",LnN(ln(50),ln(2)));
  Date("Date Estimate Peck No 1");
 };
 Boundary("End Peck No 1");
};
Sequence()
{
 Boundary("Start Jonas Field");
 Phase("Jonas Field")
 {
  R_Date("Beta-101492",590,60)
  {
   Outlier("Charcoal",1);
  };
  R_Date("Beta-85304",680,50)
  {
   Outlier("Charcoal",1);
  };
  R_Date("Beta-85305",780,60)
```

```
   {
    Outlier("Charcoal",1);
   };
  Interval("Interval Jonas Field",LnN(ln(50),ln(2)));
  Date("Date Estimate Jonas Field");
 };
 Boundary("End Jonas Field");
};
Sequence()
{
 Boundary("Start Reckner");
 Phase("Reckner")
 {
  R_Combine("Feature 77")
  {
   Outlier("General",0.05);
   R_Date("AA-53662",599,43)
   {
    Outlier("SSimple",0.05);
   };
   R_Date("AA-53662b",529,40)
   {
    Outlier("SSimple",0.05);
   };
  };
  R_Date("AA-53676",583,34)
  {
   Outlier("General",0.05);
  };
  Interval("Interval Reckner",LnN(ln(50),ln(2)));
  Date("Date Estimate Reckner");
 };
 Boundary("End Reckner");
};
Sequence()
{
 Boundary("Start Railroad Late");
 Phase("Railroad Late")
 {
  R_Date("Beta-104126",510,50)
  {
   Outlier("Charcoal",1);
  };
  R_Date("Beta-104127",650,60)
  {
   Outlier("Charcoal",1);
```

```
   };
   R_Date("Beta-104132",550,60)
   {
    Outlier("Charcoal",1);
   };
   R_Date("Beta-96146",550,90)
   {
    Outlier("Charcoal",1);
   };
   Interval("Interval Railroad Late",LnN(ln(50),ln(2)));
   Date("Date Estimate Railroad Late");
  };
  Boundary("End Railroad Late");
 };
 Sequence()
 {
  Boundary("Start Peck No 2 Late");
  Phase("Peck No 2 Late")
  {
   R_Date("AA-53309",364,44)
   {
    Outlier("Charcoal",1);
   };
   R_Date("AA-52967",321,41)
   {
    Outlier("Charcoal",1);
   };
   //R_Date("AA-52968",561,42){Outlier("Charcoal",1);};
   R_Date("AA-52969",418,42)
   {
    Outlier("Charcoal",1);
   };
   Interval("Interval Peck No 2 Late",LnN(ln(50),ln(2)));
   Date("Date Peck No 2 Late");
  };
  Boundary("End Peck No 2 Late");
 };
 Order("Order Sites");
};
};
```
